# High-Throughput Screening of a Promoter Library Reveals New Persister Mechanisms in Escherichia Coli

**DOI:** 10.1128/spectrum.02253-21

**Published:** 2022-02-23

**Authors:** Sayed Golam Mohiuddin, Aslan Massahi, Mehmet A. Orman

**Affiliations:** a Department of Chemical and Biomolecular Engineering, University of Houston, Houston, Texas, USA; University of Guelph

**Keywords:** antibiotics, bacterial proton motive force, *Escherichia coli*, lipopolysaccharide glucosyltransferase, membrane proton gradient, persister cells

## Abstract

Persister cells are a small subpopulation of phenotypic variants that survive high concentrations of bactericidal antibiotics. Their survival mechanisms are not heritable and can be formed stochastically or triggered by environmental stresses such as antibiotic treatment. In this study, high-throughput screening of an Escherichia coli promoter library and subsequent validation experiments identified several genes whose expression was upregulated by antibiotic treatment. Among the identified genes, *waaG*, *guaA,* and *guaB* were found to be important in persister cell formation in E. coli as their deletion significantly enhanced the sensitivity of cells to various antibiotics. The GuaA and GuaB enzymes form the upstream reactions of ppGpp (a global persister molecule) biosynthesis, and the deletion of *guaA* and *guaB* drastically perturbs the ppGpp regulon in E. coli. WaaG, a lipopolysaccharide glucosyltransferase, plays an important role in shaping the outer membrane structure, and the deletion of *waaG* dissipates the proton gradient (ΔpH) component of cellular proton motive force (PMF), perturbs cellular ATP production, and reduces type I persister formation in stationary phase. Active respiration in the stationary phase, which drives the PMF, was previously shown to play a critical role in type I persister formation, and our results associated with the *waaG* deficient strain further corroborate these findings.

**IMPORTANCE** Persistence is a nonheritable trait by which normal growing cells switch phenotypically to antibiotic tolerant persister cells. This transient state enables persister cells to recover and grow into an antibiotic-sensitive population. Persister cells have been observed in many pathogenic and nonpathogenic bacteria. Previous studies highlight the complexity and diversity of bacterial persister-cell mechanisms, many of which still remain to be elucidated. Here, using promoter and knockout cell libraries in Escherichia coli, we have identified genes that reveal novel persister mechanisms. As persistence is a critical survival strategy that evolved in many bacteria, our study will enhance the current molecular-level understanding of this conserved mechanism.

## INTRODUCTION

Persister cells are a small subpopulation of phenotypic variants in a bulk cell population that are reversibly tolerant to bactericidal antibiotics (1, 2). These cells represent transiently nongrowing or slow-growing cells whose survival is associated with reduced cellular activities in the targets of the antibiotics but not with mutations (3). Both stochastic and deterministic factors underlie the persister cell state, revealing the complexity and heterogeneity of persister phenotypes in prokaryotic cells (4). Some persister variants may form stochastically due to fluctuations in the levels of persistence molecules (5–7), while some variants can be induced by environmental signals (8–11). For instance, type I persisters, which are largely formed by passage through stationary phase before antibiotic treatment (12), can be induced by metabolic stresses (e.g., reactive oxygen species), overpopulation, and depletion of nutrients (9, 13, 14). These variants exhibit negligible growth after their inoculation into a fresh medium and can be highly abundant (depending on the stationary-phase inoculum size) and often tolerant to a wide range of antibiotics (3, 12, 15). We have shown that respiration in stationary-phase cells enhances intracellular degradation (i.e., self-digestion) inducing type I persisters; conversely, inhibition of stationary-phase respiration drastically reduces self-digestion and type I persisters (15–17).

Pretreatment with subinhibitory concentrations of bactericidal antibiotics increases persister levels for various bacteria, including Staphylococcus aureus (18), Klebsiella pneumoniae (19), and Staphylococcus saprophyticus and Escherichia coli uropathogens (20). However, lethal concentrations of bactericidal antibiotics can also induce persister survival mechanisms. Two studies demonstrated that fluoroquinolone-induced damage in persister cells is indistinguishable from damage seen in antibiotic-sensitive subpopulations (21, 22). The SOS response is induced in fluoroquinolone-treated cultures (21, 22), and deletion of the SOS response regulator RecA reduces persistence (21, 23, 24). RecA catalyzes DNA strand exchange reactions in the repair of double-strand breaks and stimulates the autocleavage and dissociation of the LexA repressor leading to induction of the SOS genes that repair DNA damage (25, 26). Volzing and Brynildsen showed that RecA is essential for persister recovery after the removal of antibiotics (21). The SOS response also enhances the expression of toxin protein TisB, which can arrest growth in fluoroquinolone-treated cells by decreasing proton motive force (PMF) (23).

The SOS response may be the best characterized antibiotic-induced persistence mechanism (21–24, 27), but persister mechanisms in cells induced by fluoroquinolones or other bactericidal antibiotics with different mechanisms of action (such as β-lactamases or aminoglycosides) still remain to be explored. In a library of E. coli promoters fused to a reporter, we identified several promoters that were activated by a β-lactam (ampicillin) and a fluoroquinolone (ofloxacin); however, an aminoglycoside (gentamicin) did not affect the promoters. Our subsequent assays verified that, among the genes associated with the identified hit promoters, *waaG*, *guaA* and *guaB*, were found to be involved in persister formation in E. coli.

## RESULTS

### Identifying antibiotic-induced genes using a promoter library.

The antibiotic-induced expression of genes in antibiotic-sensitive and persister cells is important in persistence. To identify such genes, we screened an E. coli K-12 MG1655 library with more than 1900 promoters fused to a fast-folding green fluorescent protein (GFP) gene in a low-copy-number plasmid (28) allowing accurate and reproducible measurement of gene expression (29). To screen the promoter library, the strains were grown in 96-well plates for 5 h to reach the early stationary phase (Fig. S1) and, then, treated with 200 μg/mL ampicillin (15) (a cell wall biosynthesis inhibitor), 5 μg/mL ofloxacin (30) (a DNA damaging agent), or 50 μg/mL gentamicin (31) (protein synthesis inhibitor) (32) for 5 h. Changes in GFP were measured hourly with a plate reader. We used metabolically active, early stationary-phase cells (Fig. S1), which provide reliable GFP measurements due to their high cell densities. Early stationary-phase cells are not easily lysed or killed by antibiotics, eliminating variations in cell density between treated and untreated cultures. In contrast, GFP levels in exponential-phase cultures of low cell density are less reliable. Because these cells are readily lysed or killed by antibiotics, GFP levels may be lower in antibiotic-treated cells compared to untreated cells, even if GFP was induced by the antibiotic. Such discrepancies in exponential-phase cultures cannot be detected easily using a plate reader.

**FIG 1:**
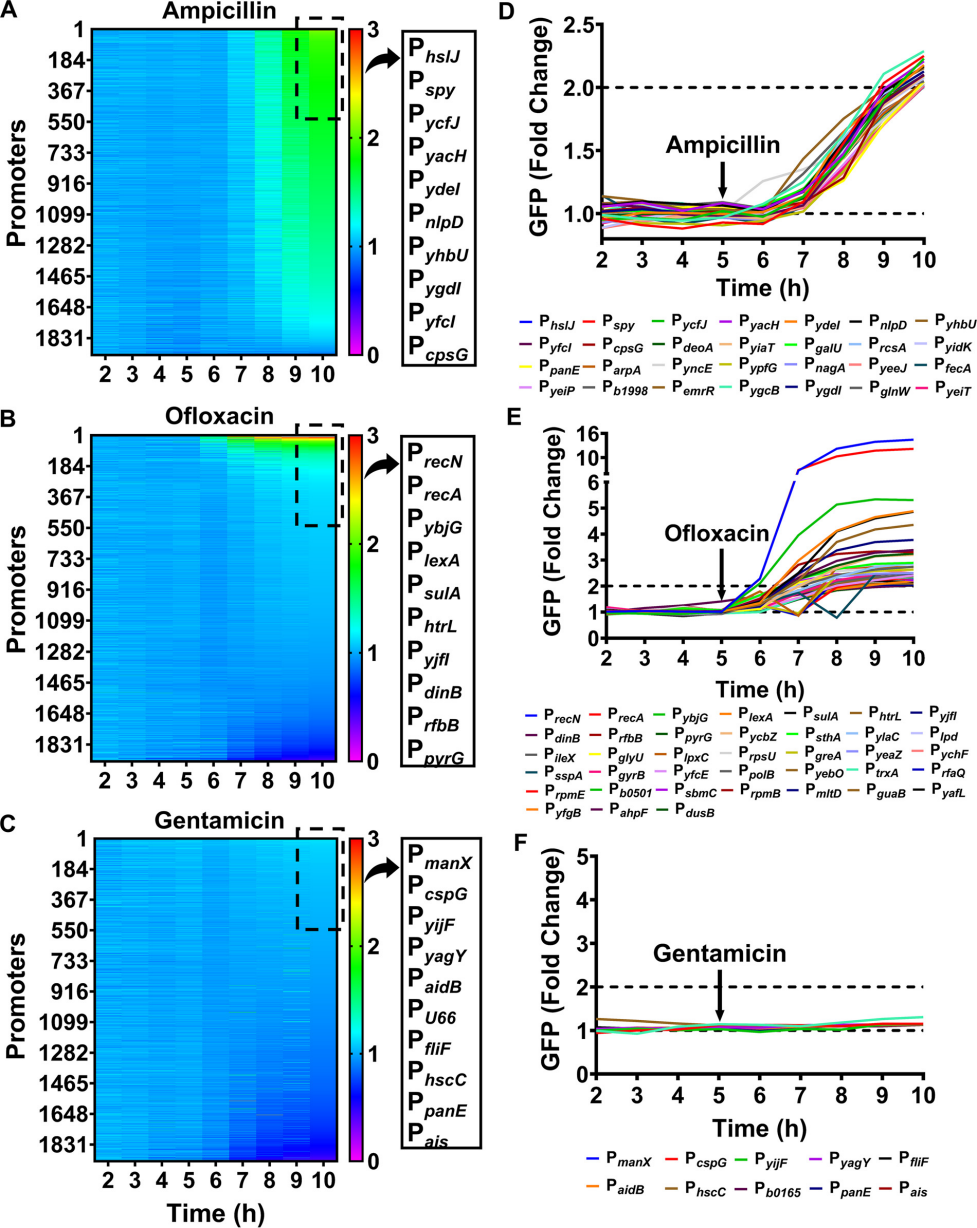
High-throughput screening of the Escherichia coli promoter library. A library of E. coli MG1655 strains with promoter reporters was treated in the early stationary phase with ampicillin (200 μg/mL), ofloxacin (5 μg/mL), or gentamicin (50 μg/mL) for 5 h (see Materials and Methods). GFP was measured at the designated times. (A–C) Fold changes for GFP (treated/untreated cultures) were used to generate heatmaps. (D–F) Temporal profiles of GFP expression in antibiotic-treated cultures for the indicated promoters. The dashed line at y = 1.0 represents data in which the GFP levels of treated and untreated cultures were the same. A 2–fold change in GFP expression (the dashed line at y = 2.0) was used as a threshold for subsequent promoter analysis. The number of biological replicates, N = 1.

We found that 28 promoters regulating the expression of ∼50 genes showed at least a 2-fold increase in GFP expression in response to ampicillin treatment (Fig. 1A and D and Table S1A), and 38 promoters regulating ∼55 genes showed at least a 2-fold increase in GFP expression in response to ofloxacin treatment (Fig. 1B and E and Table S1B). However, no promoters responded to gentamicin treatment compared to the controls (Fig. 1C and F). Ampicillin induced promoters for genes associated with the cell envelope (e.g., *galU*), membrane lipoproteins (e.g., *hslJ* and *ygdI*), outer membrane proteins (e.g., *yiaT* and *yacH*), and outer membrane transporters (e.g., *fecA*) (Table S1A). Several of the promoters that responded positively to ofloxacin treatment were related to the SOS response and DNA repair pathways (e.g., *recA*, *recN*, *polB*, *sulA*, and *dinB*) (Table S1B). We also identified ofloxacin-responsive genes for ribosomal subunits (e.g., *rpmE*, *rpsU*, and *rpmB*) and inner membrane protein (e.g., *ylaC*) (Table S1B). The detection of SOS responsive genes in ofloxacin-treated cultures validates our assay.

The high-throughput screening results for several promoters were verified at the single-cell level by flow cytometry. These included upregulated promoters P*_hslJ_* and P*_ydcS_* in ampicillin-treated cultures and P*_ybjG_* and P*_fis_* in ofloxacin-treated cultures, and unchanged promoters P*_ilvI_* and P*_argC_* in ampicillin-treated cultures and P*_yaaA_* and P*_asnC_* in ofloxacin-treated cultures (Fig. S2). Flow cytometry using these strains treated with antibiotics under the same conditions used for the screening assay provided results similar to that assay (Fig. S2), further indicating the reliability of our screening strategy.

### Identifying genes critical for bacterial persistence.

**FIG 2:**
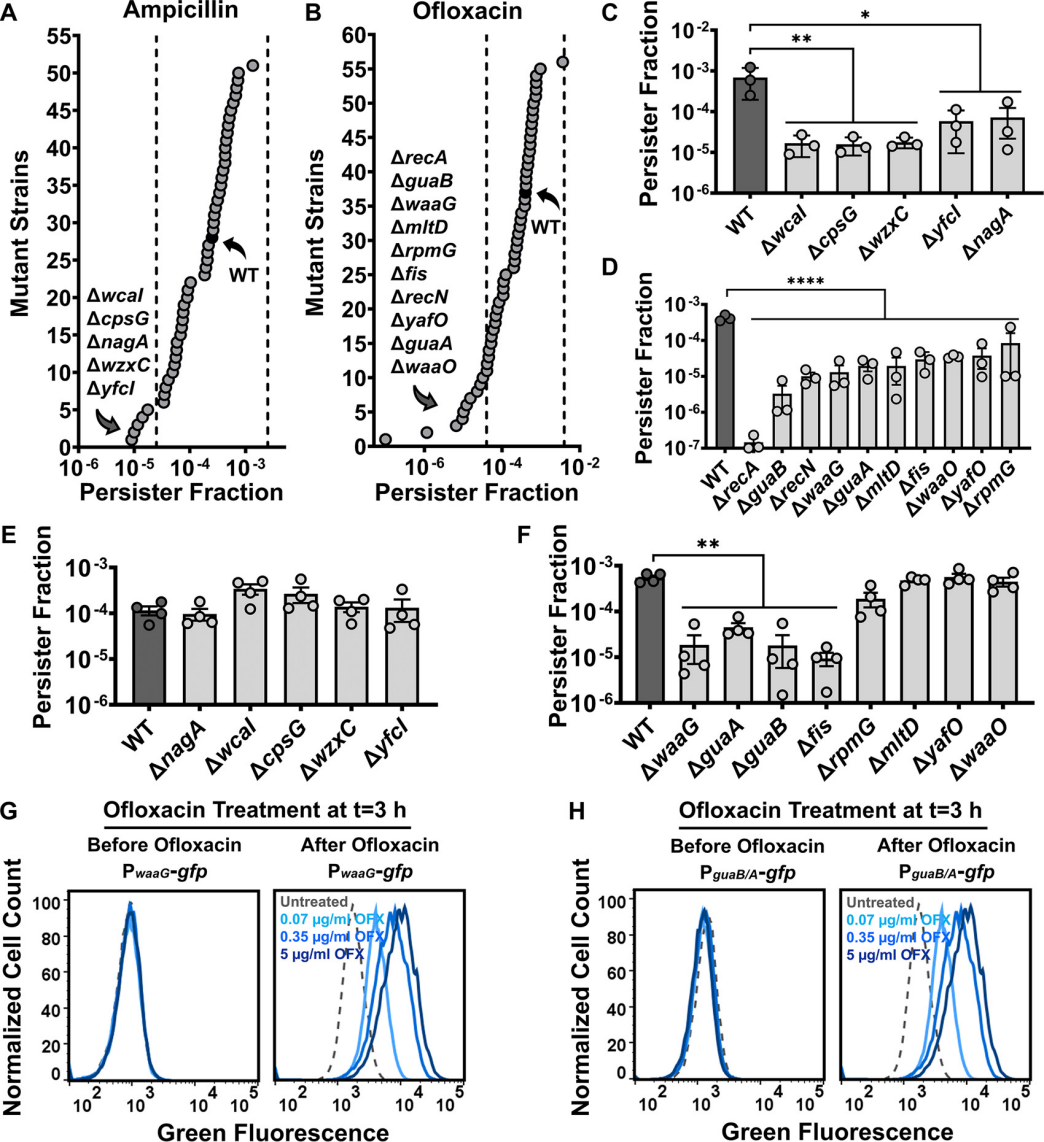
Identification of genes critical for bacterial persistence. (A–B) Persister fractions of exponential-phase cultures of E. coli BW25113 WT and selected mutant strains treated with ampicillin or ofloxacin for 5 h. A 10-fold change in persister levels was used as the threshold (dashed lines) for identifying candidate genes. N = 1. Persister fractions were calculated by taking the ratio of the number of survived cells (after treatment) to the initial number of cells (before treatment). (C–D) Persister fractions of the E. coli BW25113 mutant strains identified from panels A and B were further verified with three independent biological replicates. N = 3. (E–F) Persister fractions were determined in exponential-phase cultures of E. coli MG1655 WT and the mutant strains treated with ampicillin (E) or ofloxacin (F) for 5 h. N = 4. (G–H) GFP expression from the selected promoters was measured by flow cytometry in exponential-phase E. coli MG1655 cells with the reporter plasmids after treatment with ofloxacin (0, 0.07, 0.35, and 5 μg/mL) for 5 h. A representative replicate is shown, but similar data were obtained for all biological replicates. N = 4. For pairwise comparisons, one-way ANOVA with Dunnett’s *post hoc* test was used where *, *P* < 0.05, **, *P* < 0.01, and ****, *P* < 0.0001. Data points represent mean ± standard deviation (SD).

To determine whether loss of genes identified in the promoter screen may impair persister formation, we screened the E. coli BW25113 Keio knockout collection for mutants in the genes associated with the expressed promoters (Fig. 2A and B). Since early stationary-phase cells are intrinsically tolerant to antibiotics (especially ampicillin), we treated mid-exponential-phase cells (*t* = 3 h) with ampicillin or ofloxacin. We determined the effects of loss of 50 genes on ampicillin persistence and 55 genes on ofloxacin persistence (Table S1A and S1B). Several mutants showed at least a 10-fold reduction in persisters compared to the wild-type (WT) strain, but no mutants showed increased ampicillin- or ofloxacin-induced persistence (Fig. 2A and B). While Δ*nagA*, Δ*wcaI*, Δ*cpsG*, Δ*wzxC*, and Δ*yfcI* mutant strains have reduced ampicillin persistence, Δ*recA*, Δ*guaB*, Δ*waaG*, Δ*rpmG*, Δ*mltD*, Δ*recN*, Δ*fis*, Δ*yafO*, Δ*guaA*, and Δ*waaO* mutant strains have reduced ofloxacin persistence in E. coli BW25113 (Fig. 2C and D).

Genes whose loss reduced persister levels at least 10-fold compared to the WT strain in the Keio collection screening were deleted in E. coli MG1655 to determine the reproducibility of the persistence phenotype. Since the promoter library assay identified SOS genes (*recA* and *recN*) (Fig. 1B and E and Table S1B), and loss of these genes is known to reduce fluoroquinolone persistence (21, 27), we did not explore these genes in E. coli MG1655 further. Also, the deletion of Fis protein, a transcription factor that regulates the expression of nucleoid structure modifiers (33, 34), reduced ofloxacin persister levels in E. coli (Fig. 2D and F), as reported elsewhere (35, 36). Fis forms topological barriers that block supercoiling (37), which can rescue the cells from the damage caused by fluoroquinolone-bound gyrases (14). Although the persistence mechanism of Fis protein is well characterized (14), the ability of ofloxacin to induce *fis* expression (Fig. S2C) indicates that drug-mediated epigenetic alterations may provide alternative pathways for cells to escape death.

**FIG 3:**
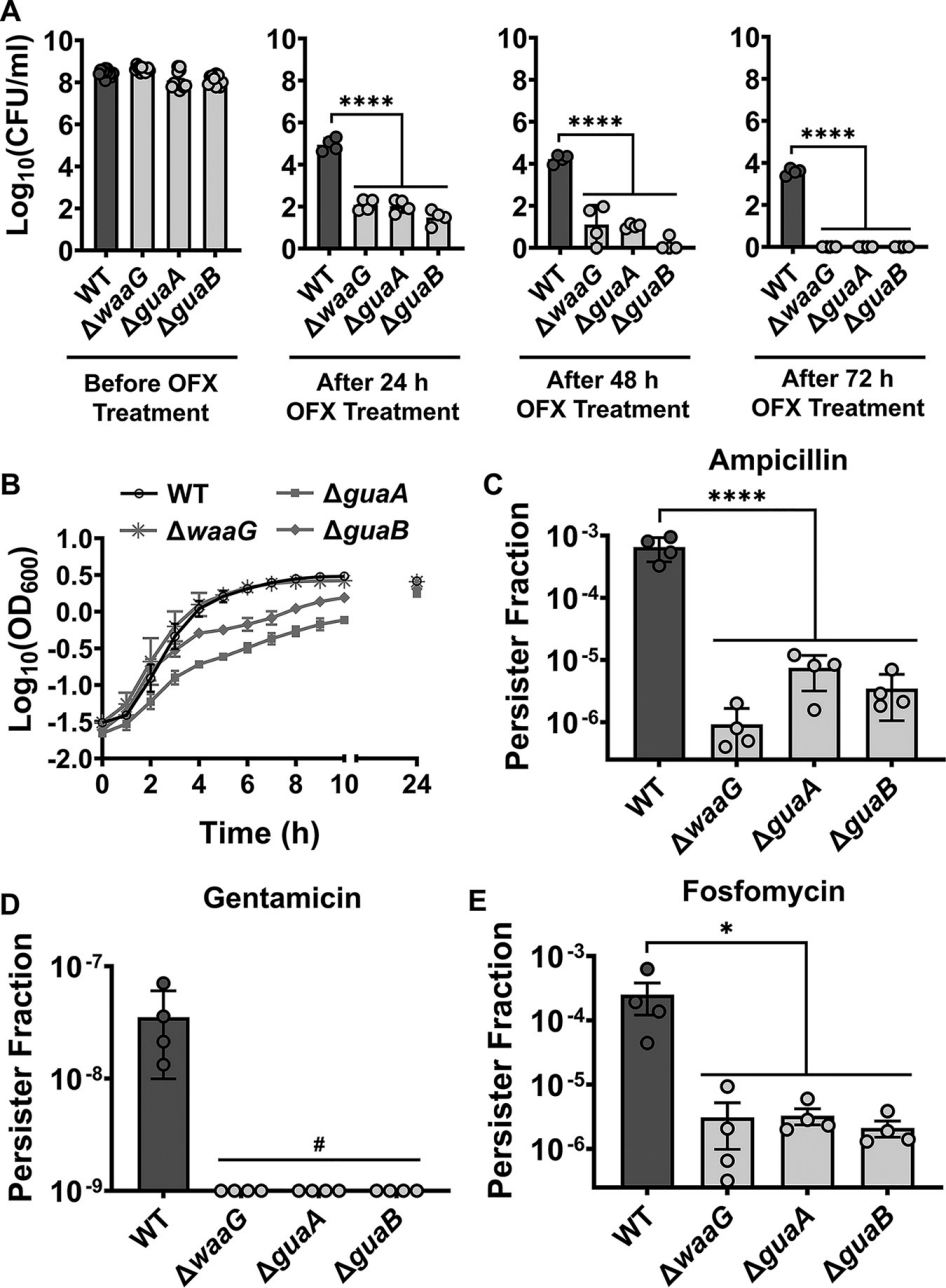
Susceptibility of mutants to antibiotics. (A) Exponential-phase E. coli MG1655 WT, Δ*waaG*, Δ*guaA*, and Δ*guaB* cells were treated with ofloxacin (5 μg/mL) for 72 h. At indicated time points (*t* = 24 h, 48 h, and 72 h), treated cells were collected and plated to enumerate colony forming units (CFU). N = 4. (B) Growth of E. coli MG1655 WT, Δ*waaG*, Δ*guaA*, and Δ*guaB* strains. OD_600_ represents the optical density measured at a wavelength of 600 nm. N = 4. (C–E) Persister levels in exponential-phase WT, Δ*waaG*, Δ*guaA*, and Δ*guaB* cells treated with ampicillin (200 μg/mL), gentamicin (50 μg/mL), or fosfomycin (300 μg/mL) for 5 h. ^#^Gentamicin persister levels of the mutant strains are under the limit of detection. N = 4. For pairwise comparisons, one-way ANOVA with Dunnett’s *post hoc* test was used where *, *P* < 0.05, **, *P* < 0.01, and ****, *P* < 0.0001. Data points represent mean ± standard deviation (SD).

In E. coli MG1655, none of the mutants identified in the ampicillin screening reduced the ampicillin persister levels significantly (Fig. 2E). However, Δ*waaG*, Δ*guaA*, and Δ*guaB* strains had significantly reduced levels of ofloxacin persisters in both strains (Fig. 2D and F). In exponential-phase cells, ofloxacin still induced GFP expression from promoters P*_waaG_* and P*_guaB/A_* (which controls both *guaA* and *guaB*) (Fig. 2G and H). Antibiotic treatment (5 μg/mL ofloxacin) for 5 h was sufficient to obtain biphasic kill curves for WT, Δ*waaG*, Δ*guaA*, and Δ*guaB* strains (Fig. S3). Moreover, longer treatment with ofloxacin (up to 72 h) caused a drastic difference between persister levels of the WT and the mutant strains (Fig. 3A). In fact, the 72-h ofloxacin treatment completely eradicated persister cells of Δ*waaG*, Δ*guaA*, and Δ*guaB* strains, while it slightly reduced the persister levels of WT (Fig. 3A).

**FIG 4:**
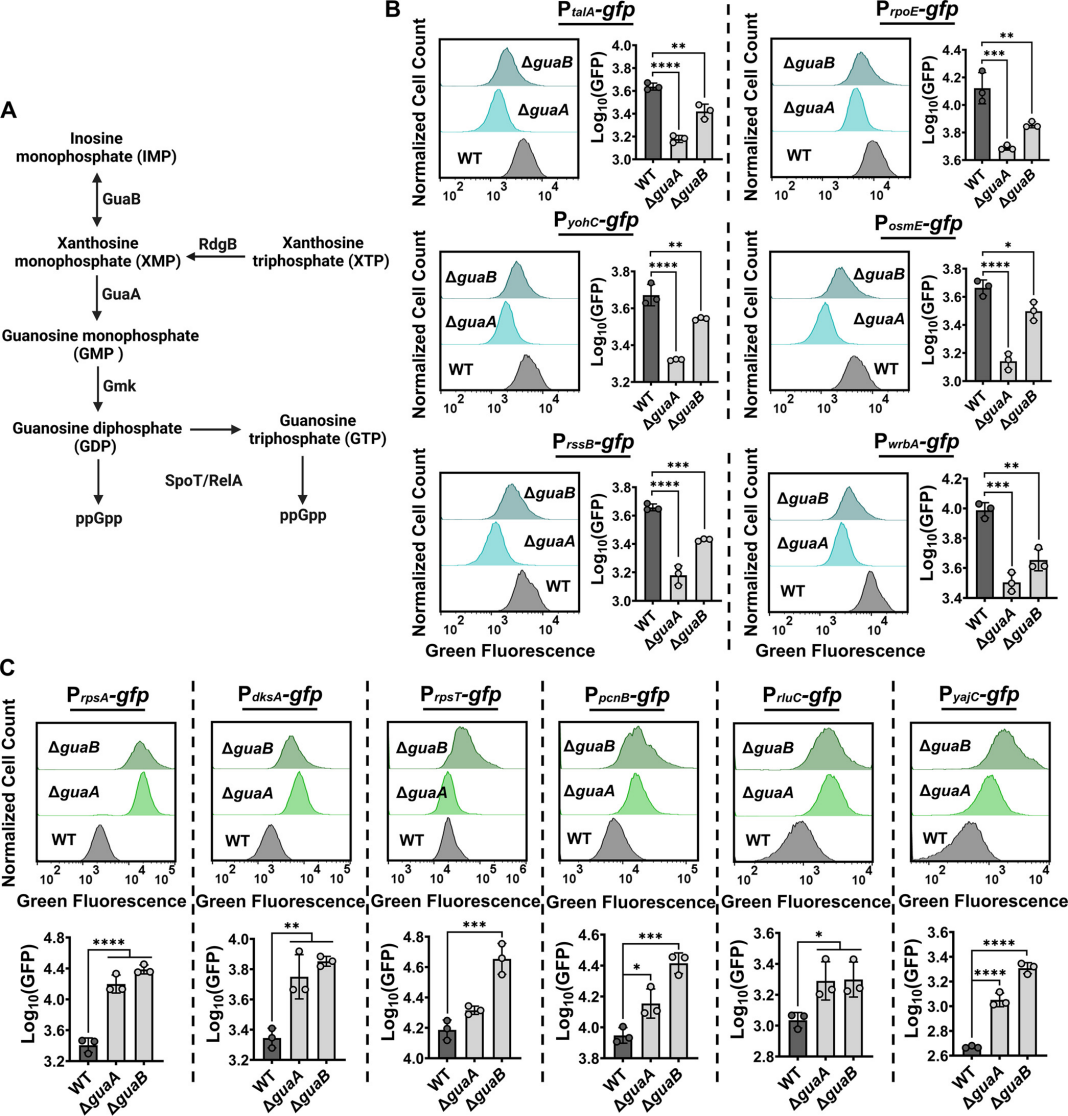
Deletions of *guaA* and *guaB* perturbed the ppGpp regulon. (A) Upstream biochemical reactions of the ppGpp biosynthesis pathway. (B-C) Stationary phase cells of WT Δ*guaA*, and Δ*guaB* strains carrying ppGpp reporters were analyzed with flow cytometry. P*_talE_*, P*_rpoE_*, P*_yohC_*, P*_osmE_*, P*_rssB_*, and P*_wrbA_* promoters are positively regulated by ppGpp (B). P*_rpsA_*, P*_dksA_*, P*_rpsT_*, P*_pcnB_*, P*_rluC_*, and P*_yajC_* promoters are negatively regulated by ppGpp (C). Mean GFP values of the WT and the mutant strains were plotted for statistical comparison. N = 3. For pairwise comparisons, one-way ANOVA with Dunnett’s *post hoc* test was used where *, *P* < 0.05, **, *P* < 0.01, ***, *P* < 0.001, and ****, *P* < 0.0001. Data points represent mean ± standard deviation (SD).

Deletion of *guaA* and *guaB* significantly impaired cell growth (Fig. 3B) and all these mutants were sensitive to ampicillin (200 μg/mL), gentamicin (50 μg/mL), and fosfomycin (300 μg/mL) (Fig. 3C to E). Although this broad antibiotic sensitivity suggests that the reduced persister levels in Δ*waaG*, Δ*guaA*, and Δ*guaB* mutants are not antibiotic specific, the mechanisms of action likely differ since the proteins encoded by these genes affect unrelated cellular processes. While WaaG is lipopolysaccharide glucosyltransferase I, a member of glycosyltransferase family enzymes (38), GuaA (guanine monophosphate synthetase) and GuaB (inosine 5′-monophosphate dehydrogenase) synthesize guanine monophosphate from inosine monophosphate (39, 40) and comprise the upstream reactions of the ppGpp biosynthesis pathway (Fig. 4A).

### Deletions of *guaA* and *guaB* perturbed the ppGpp regulon.

The GuaA and GuaB enzymes are crucial for the maintenance of the guanine nucleotide pool (39–41). Although the perturbation of *guaA* and *guaB* enhanced Bacillus subtilis persistence (42), we presume that the deletion of these genes reduces persister levels by compromising the ppGpp biosynthesis in E. coli (14). DksA protein together with ppGpp (ppGpp-DksA) form a global regulator of the stringent response, and the disruption of the ppGpp regulon has been repeatedly shown to impair E. coli persistence (7, 14). To monitor the ppGpp regulon in Δ*guaA* and Δ*guaB* strains, we used 6 different reporter plasmids, where *gfp* was fused to promoters of *talA*, *rpoE*, *yohC*, *osmE*, *rssB*, and *wrbA* genes (P*_talE_*, P*_rpoE_*, P*_yohC_*, P*_osmE_*, P*_rssB_*, and P*_wrbA_*). These promoters, some of which have been already used to assess the ppGpp function in previous studies (14, 43, 44), are positively regulated by ppGpp (14, 43, 44). Basically, they express GFP in the presence of ppGpp; therefore, the accumulation of GFP should be increased more in WT than in mutant strains, as verified by our data (Fig. 4B). We note that this observed reduction in GFP expression in the mutant strains might be related to their slow growth; therefore, we tested 6 additional reporters containing the promoters of *rpsA*, *dksA*, *rpsT*, *pcnB*, *rluC*, and *yajC* genes (P*_rpsA_*, P*_dksA_*, P*_rpsT_*, P*_pcnB_*, P*_rluC_*, and P*_yajC_*). The ppGpp molecule is known to inhibit the binding of RNA polymerase to these selected promoters, thus negatively regulating them (44–47). Despite their growth deficiency, Δ*guaA* and Δ*guaB* cells express more GFP from these reporter plasmids than WT cells (Fig. 4C), further indicating the impaired ppGpp regulon in the Δ*guaA* and Δ*guaB* mutant strains. Of note, given that many E. coli promoters are collectively regulated by a diverse range of transcription factors, we specifically chose promoters like P*_yohC_*, P*_rpsA_*, P*_rpsT_*, P*_pcnB_*, P*_rluC_*, and P*_yajC_*, which, to the best of our knowledge, are regulated by ppGpp-DksA only (48). The data obtained from these promoters validate that the ppGpp regulator seems to be the sole factor inducing the observed translational rewiring.

### Deletion of *waaG* gene reduced ofloxacin persister formation in E. coli stationary-phase cultures.

**FIG 5:**
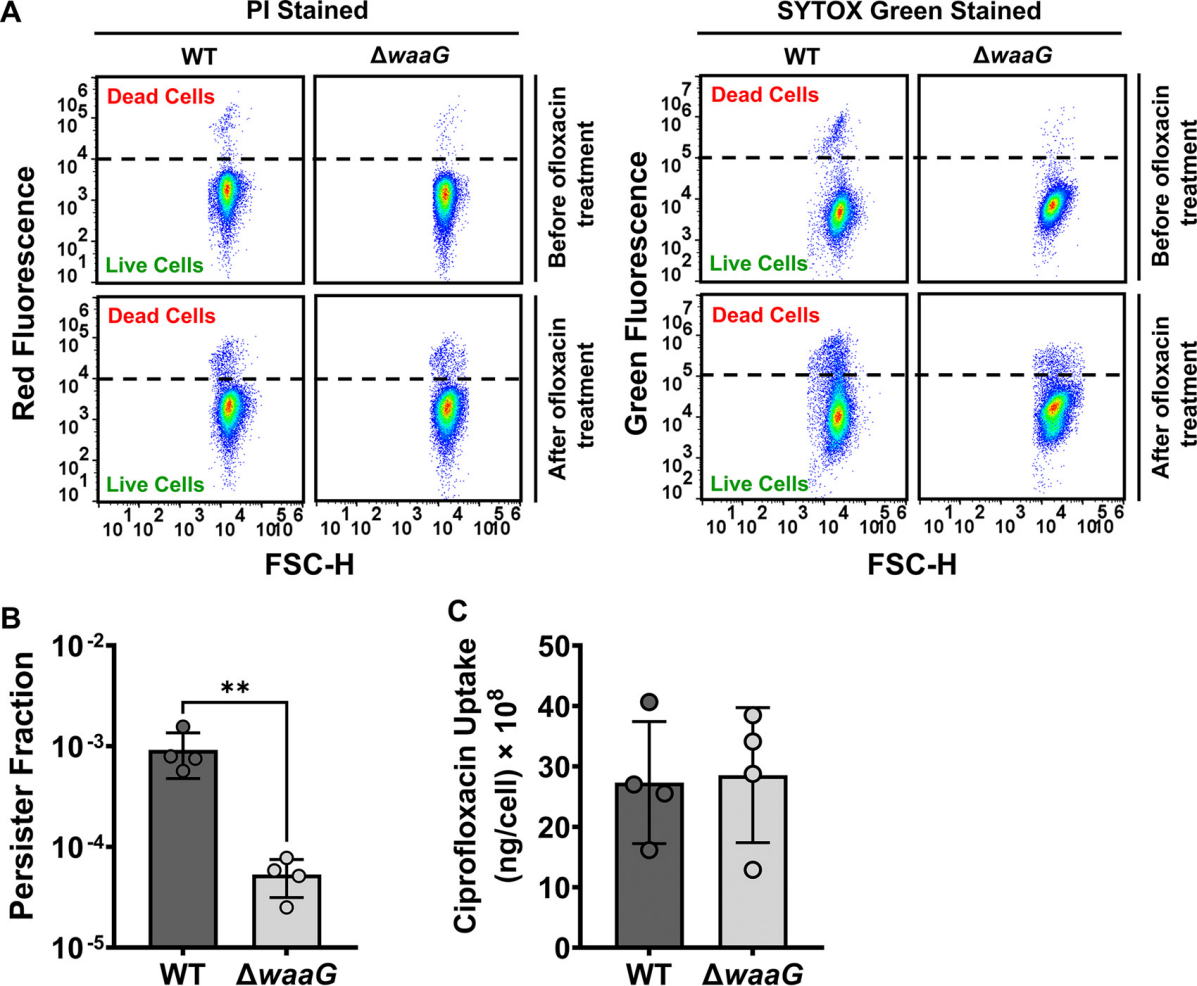
Deletion of the *waaG* gene did not increase membrane permeabilization. (A) Exponential-phase E. coli MG1655 WT and Δ*waaG* cells were treated with ofloxacin for 5 h, stained with propidium iodide (PI) or SYTOX Green, and analyzed by flow cytometry to determine the membrane permeability of the cells. Cells treated with 70% ethanol were used as a positive control. Stained live cells were used as a negative control. Positive and negative controls were used to gate live and dead cells populations (Supp. Fig. S4). A representative replicate is shown, but similar data were obtained for all biological replicates. N = 4. (B) Exponential-phase E. coli MG1655 WT and Δ*waaG* cells were treated with ciprofloxacin (3 μg/mL) for 5 h. After treatment, cells were plated to determine persister fractions. N = 4. (C) Cells after ciprofloxacin treatment were collected, washed to remove the antibiotic from the supernatant, and then, lysed with a cell lysis buffer to release the intracellular ciprofloxacin. Intracellular ciprofloxacin levels were measured with a plate reader. N = 4. Pairwise comparison was performed using a two-tailed Student's *t* test with unequal variance, where **, *P* < 0.01. Data points represent mean ± standard deviation (SD).

The WaaG glucosyltransferase plays a vital role in the outer membrane by providing the first outer core d-glucose of lipopolysaccharides (49). Although the Δ*waaG* strain grows well and reaches the mid-exponential phase (OD_600_∼0.5) around 3 h, similar to the WT strain (Fig. 3B), perturbation of this gene potentially affects membrane integrity (50, 51). This may increase membrane permeability and drug uptake, reducing persister levels in the Δ*waaG* strain. However, neither ofloxacin-treated nor untreated WT or Δ*waaG* cells showed any differences when stained with propidium iodide (PI; a red fluorescent dye) or SYTOX Green (a green fluorescent dye) (Fig. 5A). Although these two dyes are structurally different, they penetrate only cells with compromised membrane integrity. Moreover, given that ciprofloxacin exhibits a fluorescence emission at 410 nm when excited at 275 nm (52, 53), we measured intracellular ciprofloxacin levels of the WT and Δ*waaG* strains using an established method (52, 53). Although the ciprofloxacin persister level in the Δ*waaG* strain is still lower than that of WT (Fig. 5B), we found no difference in ciprofloxacin uptake between the WT and Δ*waaG* cell populations (Fig. 5C). These results, altogether, suggest that changes in membrane permeability may not be the major factor underlying the reduction in persister levels in the Δ*waaG* strain.

**FIG 6:**
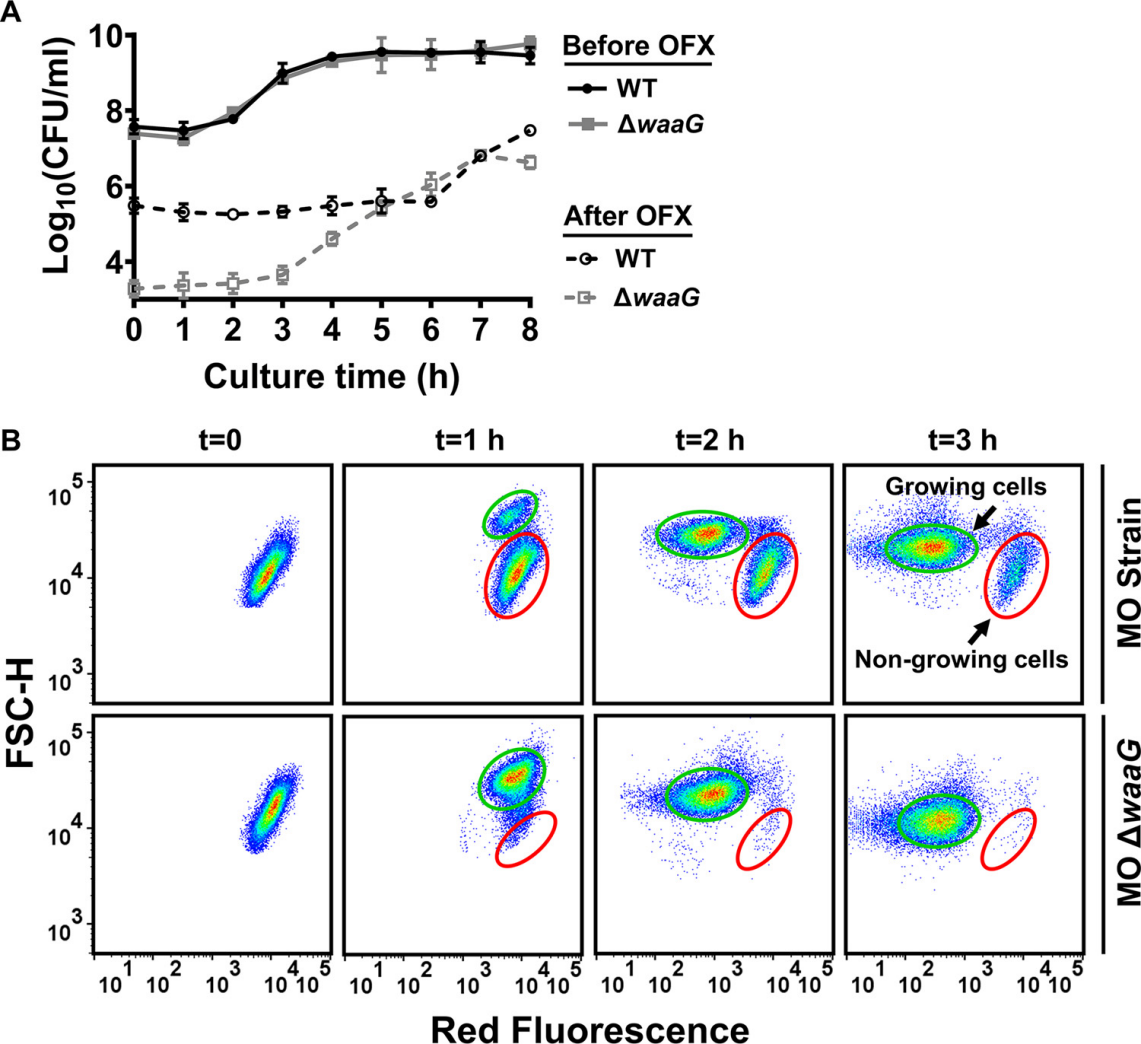
Deletion of the *waaG* gene reduced nongrowing cells in the stationary phase. (A) E. coli MG1655 WT and Δ*waaG* cells were collected at indicated times from the cultures and treated with ofloxacin. CFU levels of ofloxacin treated and untreated cultures were provided on the figure. N = 4. (B) E. coli MG1655 MO and Δ*waaG* cells expressing an isopropyl β-D-1-thiogalactopyranoside (IPTG)-inducible *mCherry* were grown with 1 mM IPTG in overnight precultures. At late stationary phase (*t* = 24 h), overnight precultures were diluted 100-fold in a medium without IPTG. At the indicated times, cells were collected and analyzed by flow cytometry. A representative replicate is shown, but similar data were obtained for all biological replicates. N = 4. Data points represent mean ± standard deviation (SD).

To determine persisters, precultures are typically diluted in fresh medium, grown to a specific growth phase, and then treated with antibiotics. In lag-phase cultures, there were significantly fewer ofloxacin persisters carried over from precultures in the Δ*waaG* culture than in the WT culture (Fig. 6A). In the precultures, the passage through the stationary phase induces a growth-inhibited cell subpopulation that exhibits a slow exit from the stationary phase after transfer to a fresh medium. This nongrowing subpopulation is enriched with type I persisters (54) and “viable but nonculturable” cells (55, 56). Stationary phase E. coli cells become small and spherical with rigid membranes (57). The number of nongrowing cells increases the longer the culture stays at the stationary phase (58). Here, we distinguished nongrowing and growing cells with a fluorescent protein dilution assay as described previously (54, 59). Precultures of cells (E. coli MG1655 MO) having an inducible *mCherry* expression system were grown with the inducer to produce mCherry protein. After the inoculation of the precultures in fresh media without the inducer, nongrowing cells were monitored by measuring mCherry levels of the cell population with flow cytometry. Growing cells have less mCherry than nongrowing cells because it is diluted by cell division (Fig. 6B). Also, nongrowing cells have reduced forward scatter (FSC) signals, which correlate with cell size. As expected, the Δ*waaG* strain showed fewer nongrowing cells compared to the parental strain (E. coli MG1655 MO) (Fig. 6B). We previously showed that there are at least 100-fold more persisters in the nongrowing cell subpopulation than the growing cell subpopulation in E. coli (54). The lack of a nongrowing cell subpopulation in Δ*waaG* (Fig. 6B) explains the observed ∼100-fold reduction in persister levels in this strain (Fig. 6A). The lack of these multidrug tolerant type I persisters in the Δ*waaG* strain can also explain the sensitivity of this strain to other antibiotics (Fig. 3C to E). Also, the presence of type I persisters in WT cultures might be the reason why the WT strain is more tolerant to longer treatments compared to the Δ*waaG* strain (Fig. 3A).

Since lipopolysaccharides play a key role in biofilm formation (60, 61) and biofilms are enriched in antibiotic-tolerant slow or nongrowing cells (62), we determined whether biofilms of the Δ*waaG* strain were more susceptible to antibiotics than WT biofilms. Cells were grown on polyethersulfone (PES) membrane on LB agar plates for 24 h and then transferred to LB plates with ofloxacin for quantitation of persister cells (63). The Δ*waaG* biofilm had 100-fold fewer ofloxacin persister cells than the WT biofilm, which accounts for its greater susceptibility to antibiotics (Fig. S5).

### Deletion of *waaG* dissipates the membrane proton gradient in the stationary phase.

**FIG 7:**
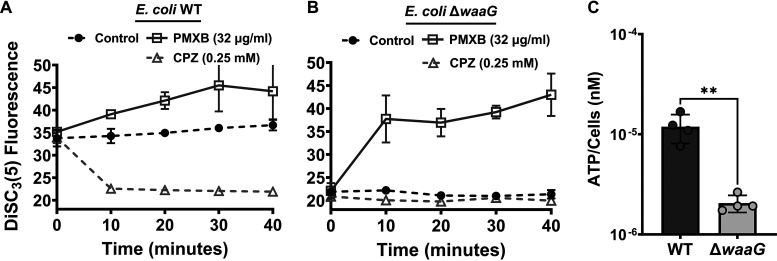
Deletion of the *waaG* gene reduced PMF and intracellular ATP levels. (A–B) Late stationary-phase (*t* = 24 h) cells of WT and Δ*waaG* strains in overnight precultures were diluted and stained with DiSC_3_(5) (see Materials and Methods). After reaching equilibrium, the stained cells were treated with polymyxin B or chlorpromazine (CPZ) at the indicated concentrations. At the indicated times, fluorescence was measured with a plate reader. N = 4. (C) At late stationary phase (*t* = 24 h) in overnight precultures, the ATP levels in WT and Δ*waaG* cells were measured. N = 4. Pairwise comparison was performed using a two-tailed Student's *t* test with unequal variance. **, *P* < 0.01. Data points represent mean ± standard deviation (SD).

Active respiration in the stationary phase aids in the arrest of growth in some cells (13, 15) by inducing metabolic stress such as self-digestion (15). Inhibition of stationary-phase respiration, which drives the PMF, reduces the number of nongrowing cells (13, 15) and type I persisters (15). Maintenance of the PMF was also found to be essential for starvation-induced antibiotic-tolerant cells (64). Although exponentially growing cells of the Δ*waaG* strain showed no change in membrane permeability, the *waaG* mutation affects membrane structure (50), which is important for maintaining the PMF (65). To determine whether the PMF was impaired in the Δ*waaG* strain in the stationary phase, we used a membrane potential sensitive dye, 3,3′-dipropylthiadicarbocyanine iodide [DiSC_3_(5)] (65, 66) to measure dissipation of the electrical potential (ΔΨ) and the proton gradient (ΔpH) components of the PMF (65, 66). The DiSC_3_(5) assay was adapted from a previous study (66, 67) and is based on the fact that when the ΔΨ component of the PMF is dissipated, the probe is released into the medium, increasing fluorescence (66, 67). However, the dissipation of the ΔpH component results in a decrease in fluorescence (65–67). This is because the dissipation of ΔpH is compensated for by an increase in ΔΨ, which stimulates the accumulation of the probe in the cell membrane. DiSC_3_(5) quenches itself upon accumulation in the cell membrane. The fluorescence of the Δ*waaG* strain was significantly lower than the WT strain suggesting selective dissipation of ΔpH in the mutant strain (Fig. 7A and B). As expected, the dissipation of the ΔpH also reduced intracellular ATP levels in stationary-phase Δ*waaG* cells (Fig. 7C). As a control, we treated the DiSC_3_(5)-stained cells with polymyxin B, a cationic polypeptide that dissipates ΔΨ (66) and chlorpromazine (CPZ), an ATP synthase inhibitor (Fig. S6) (68, 69) that dissipates ΔpH. While CPZ reduced the fluorescence of WT cells, it did not alter the fluorescence of the Δ*waaG* cells (Fig. 7A and B) since the ΔpH component of the PMF is already dissipated in this mutant. Similarly, treating the stationary-phase precultures with CPZ (used as control) greatly reduced nongrowing cells (Fig. S7) and ofloxacin persister cell levels (Fig. S8), consistent with a published study (17). Overall, our study further demonstrates the importance of the PMF in persister cell formation in stationary-phase cultures.

## DISCUSSION

Persistence, which occurs in many eukaryotic and prokaryotic cell types, allows organisms to survive unfavorable environmental conditions. Persister cells can form stochastically due to random fluctuations in levels of persister molecules, e.g., ppGpp, and toxins (5–7). However, deterministic mechanisms triggered by environmental signals such as antibiotic treatment can also affect the level of persister cells in the population (8–10). Fluoroquinolones induce the SOS response in both antibiotic sensitive and tolerant cells, and SOS regulatory and repair proteins are essential for the recovery of persister cells (21, 24). Although the induction of SOS genes (e.g., *recA*) in antibiotic-sensitive cells does not necessarily rescue them, these genes are essential for the maintenance of fluoroquinolone persistence (21). The growth-arrest mechanism, the level of cellular damage, and the extent of repair may collectively determine the fate of each cell in antibiotic-treated cultures.

To find additional genes or pathways that generate persister cells, we screened a library of E. coli promoter strains by inducing with three antibiotics (ampicillin, ofloxacin, and gentamicin) that have different mechanisms of action. Although we observed a huge variation in the expression profiles of reporter plasmids in the ofloxacin-treated cultures, less variation was observed in the ampicillin-treated cultures compared to the ofloxacin-treated cultures and not a lot of promoters responded to gentamicin treatment. Cellular response to antibiotics may depend on the mechanism of action of the antibiotic used as well as the cytotoxic molecules produced by the treatment. For example, ofloxacin inhibits DNA gyrase during DNA replication; this leads to DNA breaks that drastically enhance the expression of SOS response-related genes (26), as reported in this study.

Persistence is highly sensitive to culture conditions and strains used; therefore, different outcomes can be expected for different cell types (Fig. 2C and E), growth phases (Fig. 6A), and treatment durations (Fig. 3A). Although we did not determine whether the identified genes might collectively affect E. coli persistence, ampicillin, which targets cell wall synthesis, induced promoters for genes associated with the cell envelope, membrane lipoproteins, outer membrane proteins, and outer membrane transporters. Interestingly, none of the gene deletions identified in the ampicillin screening of E. coli BW25113 mutants reduced the persister levels of E. coli MG1655, indicating the existence of different persistence mechanisms across E. coli strains. The persister levels of BW25113 Δ*rpoS* and Δ*ihfA* mutant strains were already shown to be different than those of other E. coli species in previous studies (70–72). E. coli BW25113, the derivative of K-12 strain, was generated by the laboratory of Barry L Wanner (73) and used as the parental strain for the Keio collection (73, 74). This strain has *araBAD* and *rhaDAB* deletions and four tandem *rrnB* terminators in the locus of *lacZ*, and the genomic sequencing of the chromosomal DNA of the BW25113 strain revealed the presence of 20 substitutions and 11 indels (75). For the scientific reproducibility, we specifically focused on the genes whose deletions reduced persistence in both E. coli MG1655 and BW25113 strains in this study.

Several of the promoters that responded positively to ofloxacin treatment were related to the SOS response, DNA repair pathways, ribosomal subunits, and inner membrane proteins. We did not study genes related to the SOS response here; however, single deletions of *guaA*, *guaB*, or *waaG* significantly reduced the ofloxacin persisters. Strains Δ*guaA* and Δ*guaB* are auxotrophic for guanine (41, 76), and both grew slowly in standard LB growth medium. However, despite their slower growth, Δ*guaA* and Δ*guaB* developed significantly fewer persister cells in response to multiple antibiotics, challenging the idea that slow growth is associated with the formation of persisters (12, 77, 78). Although the perturbation of *guaA* and *guaB* enhances Bacillus subtilis persistence, highlighting the existence of distinct persistence mechanisms across species (42), we think that the reduced persister levels in E. coli Δ*guaA* and Δ*guaB* strains may be due to the perturbation of intracellular ppGpp (39, 41, 79, 80), as GuaA and GuaB belong to the ppGpp biosynthesis pathway (80). Although GuaB synthesizes XMP from IMP, XMP can also be synthesized by deoxyribonucleoside triphosphate pyrophosphohydrolase, an enzyme encoded by the *rdgB* gene (Fig. 4A). On the other hand, GuaA, synthesizing GMP, forms the metabolic bottleneck of the ppGpp biosynthesis pathway (Fig. 4A), which may explain the severe growth deficiency observed in the Δ*guaA* strain (Fig. 3B). Loss of *guaA* and *guaB* may have other pleiotropic effects as these enzymes are crucial for the maintenance of the guanine nucleotide pool (39–41).

WaaG plays an important role in shaping the outer membrane structure. Loss of the enzymes responsible for membrane lipopolysaccharide synthesis can affect cell surface hydrophobicity, outer membrane permeability and integrity, and biofilm formation in bacteria (51). We demonstrated that the Δ*waaG* strain had significantly reduced levels of nongrowing cells in stationary-phase cultures under the conditions studied here. The persister cell percentage of the nongrowing cell subpopulation was previously shown to be much higher (∼100-fold) than that of the growing subpopulation in E. coli cells (54), consistent with the reported difference between Δ*waaG* and WT persister levels. Metabolic stresses (e.g., self-digestion) and free radicals associated with respiration in the stationary phase, damage cells and induce the formation of nongrowing cells (15, 16). We have previously shown that treatment of cells with respiration inhibitors such as potassium cyanide or CPZ, or anaerobic cell culture reduces persister-cell formation in the stationary phase (15–17). We corroborated that loss of *waaG* dissipates the ΔpH component of the PMF and reduced respiration, but the normal growth of the Δ*waaG* strain in a rich medium is perplexing. We speculate that these cells might enhance glycolysis to meet their demands for energy and other intermediate molecules required for macromolecular synthesis. A Warburg-like metabolism, i.e., aerobic glycolysis, which is characterized by increased glycolysis and decreased aerobic phosphorylation, is a metabolic strategy of fast-growing mammalian cells (e.g., cancer cells). However, whether such a mechanism functions in the Δ*waaG* strain requires further investigation.

In conclusion, persistence is a very complex, critical, survival strategy that evolved in many bacteria; thus, our study will augment the current molecular-level understanding of this conserved mechanism. Further, experimental strategies developed here will contribute significantly to the field, since many aspects of persister mechanisms in diverse organisms remain unknown, and novel and robust strategies such as these can identify these mechanisms.

## MATERIALS AND METHODS

### Bacterial strains and plasmids.

The promoter strain collection of Escherichia coli MG1655 (28) in a 96-well plate format used for the screening assay was obtained from Horizon Discovery, Lafayette, CO, USA. The Keio knockout strain collection (a single-gene deletion library of E. coli K-12 BW25113) was obtained from Horizon Discovery. The strains from the Keio knockout collection used in this study are listed in Table S2A. These strains were selected based on our promoter screening data (Fig. 1). We found that 28 promoters showed at least a 2-fold increase in GFP expression in response to ampicillin treatment, and 38 promoters in response to ofloxacin treatment. Using the EcoCyc operon database (48), we identified ∼50 nonessential genes that are regulated by the promoters detected from ampicillin treatment and ∼55 nonessential genes that are regulated by the promoters detected from ofloxacin-treatment (Table S2A).

The method of Datsenko and Wanner (73) was used to generate Δ*nagA*, Δ*wcaI*, Δ*cpsG*, Δ*wzxC*, Δ*yfcI*, Δ*guaB*, Δ*waaG*, Δ*rpmG*, Δ*mltD*, Δ*fis*, Δ*yafO*, Δ*guaA*, and Δ*waaO* mutant strains in E. coli K-12 MG1655 (Table S2B). Oligonucleotides used to delete the genes are in the Table S2C. An E. coli K-12 MG1655 (MO) strain carrying an IPTG-inducible T5 promoter and the highly expressed *lacI^q^* repressor was used to express mCherry (54). The method of Datsenko and Wanner was used to delete *waaG* from this background strain (Table S2B). Gene deletions leading to a reduction in persistence were validated with check primers in both E. coli MG1655 and BW25113 backgrounds (Table S3).

### Chemicals, media, and growth conditions.

Unless stated otherwise, all chemicals used in this study were purchased from Fisher Scientific (Atlanta, GA) or VWR International (Pittsburg, PA). PI staining and ATP measurement kits were purchased from Thermo Fisher (Waltham, MA) and Promega Corporation (Madison, WI). Standard Luria-Bertani (LB) broth was prepared by dissolving 5 g yeast extract, 10 g tryptone, and 10 g sodium chloride in 1 l deionized (DI). LB agar was prepared by dissolving 40 g of premixed LB agar powder in 1 l DI water. LB broth and LB agar were sterilized by autoclaving at 121°C and 103.421 KPa. Ampicillin (200 μg/mL), ofloxacin (5 μg/mL), gentamicin (50 μg/mL), fosfomycin (300 μg/mL), and ciprofloxacin (3 μg/mL) were used to quantify persister cells in cultures. Antibiotic concentrations for persister assays were chosen to be much higher than MICs, consistent with previous studies (30, 31, 54). MICs of antibiotics for E. coli were determined using a 2-fold dilution method as described previously (81) and tabulated in Table S4. Kanamycin at 50 μg/mL was used to maintain plasmids in the cells (54). IPTG at 1 mM was used to induce mCherry expression (15, 54). CPZ (0.25 mM) was used to inhibit bacterial PMF. Polymyxin B (32 μg/mL) was used to dissipate the electron gradient (66). Sterile 1X phosphate buffer saline (PBS) solution was used to wash the cells to reduce the antibiotic concentration below the MIC. For PI staining, sterile 0.85% sodium chloride solution was used. Antibiotics and IPTG were dissolved in DI water and sterilized with a 0.2 μm PES syringe filter. A solution of NaOH was used to dissolve the ofloxacin, as described elsewhere (82). Unless stated otherwise, overnight precultures were prepared by inoculating cells from frozen 25% glycerol (−80°C) cell stocks into 2 mL LB medium in 14-mL round-bottom Falcon tubes. After culturing 24 h at 37°C with shaking at 250 rpm, cells from the overnight precultures were diluted 100-fold in 2 mL fresh medium in 14-mL tubes and cultured to obtain the desired growth phase for assays.

### Screening the promoter library.

Overnight precultures were prepared by inoculating the strains from the promoter library into the wells of 96-well plates containing 250 μl LB medium. The plates were sealed with a sterile, oxygen-permeable membrane (Breathe-Easier, Cat# BERM-2000, VWR International) and cultured for 24 h at 37°C with shaking at 250 rpm. Overnight precultures were diluted 1:50 in LB medium in a new 96-well plate, sealed, and incubated as described above. At early stationary phase (*t* = 5 h), cells were treated with ampicillin (200 μg/mL), ofloxacin (5 μg/mL), and gentamicin (50 μg/mL) for 5 h. GFP was measured with a Varioskan LUX Multimode Microplate Reader (Thermo Fisher, Waltham, MA, USA) at the indicated times with untreated cultures as a control (see Data set S1). The excitation and emission wavelengths for GFP measurement were 485 nm and 511 nm, respectively. For validation experiments, strains with the reporter plasmids for P*_hslJ_*, P*_ydcS_*, P*_ybjG_*, P*_fis_*, P*_ilvI_*, P*_argC_*, P*_yaaA_*_,_ and P*_asnC_* promoters were grown and treated with antibiotics as described above. At indicated times, cells were diluted in PBS to a cell density of 10^6^–10^7^ cells/mL and analyzed with a flow cytometer (NovoCyte Flow Cytometer, NovoCyte 3000RYB, ACEA Biosciences Inc., San Diego, CA, United States). The cells were excited at a wavelength of 488 nm, and the green fluorescence signal was quantified with a 530/30 nm bandpass filter.

### Cell growth assay.

Overnight precultures were prepared by inoculating cells from frozen stocks into 2 mL LB medium in 14-mL round-bottom tubes. After culturing for 24 h at 37°C with shaking (250 rpm), overnight precultures were diluted 100-fold in 14-mL Falcon tubes containing 2 mL LB media and incubated in the shaker at 250 rpm at 37°C. The growth of the cultures was determined by measuring the optical density at 600 nm (OD_600_) with a plate reader.

### Persister assay.

Overnight precultures were prepared by inoculating cells from frozen stocks into 2 mL LB medium in 14-mL round-bottom tubes. After culturing for 24 h at 37°C with shaking (250 rpm), overnight precultures were diluted 100-fold in 14-mL Falcon tubes containing 2 mL LB medium and cultured in the incubator at 37°C with shaking. Cells at exponential phase (t = 3h) were treated with antibiotics at indicated concentrations for 5 h. To determine the number of live cells before antibiotic treatment, 10 μl of cell cultures were serially diluted in PBS and plated on an LB agar plate and incubated for 16 h at 37°C. During antibiotic treatments, 1 mL cultures were collected at the indicated times and washed twice with PBS by centrifugation at 13,300 rpm (17,000 g) for 3 min (min) to remove antibiotics. After the final centrifugation, 900 μl of supernatant was removed with a pipette; the cell pellets were resuspended in the remaining 100 μl PBS; 10 μl of the cell suspensions were serially diluted in PBS; and 10 μl of the diluted cell suspensions were spotted on LB agar plates. After at least 16 h at 37°C, the CFU were counted to determine the number of live cells in the cultures. Persister fractions were determined by taking the ratio of the number of survived cells (after treatment) to the initial number of cells (before treatment). We typically incubate the plates at 37°C for 72 h to assess whether a longer incubation period is necessary for the colony formation of mutant cells. Although the existing colonies grew larger with longer incubation times, the observed difference in CFU between the WT and mutant strains did not change with longer incubations (Fig. S9).

### Measuring the activities of the P*_waaG_* and P*_guaB/A_* promoters.

Overnight precultures of E. coli strains with reporter plasmids for P*_waaG_* and P*_guaB/A_* were diluted 100-fold in 14-mL Falcon tubes containing 2 mL LB broth and cultured at 37°C with shaking. At the exponential phase (*t* = 3), cells were treated with ofloxacin or ampicillin for 5 h. Before and after the treatment, cells were diluted in PBS to 10^6^–10^7^ cells/mL and GFP was determined by flow cytometry. The cells were excited at a wavelength of 488 nm, and the green fluorescence signal was quantified with a 530/30 nm bandpass filter.

### Measuring the activities of ppGpp regulated promoters.

Overnight cultures of WT, Δ*guaA*, and Δ*guaB* strains harboring the ppGpp reporters (i.e., promoter plasmids that are regulated by ppGpp) were prepared by inoculating cells from frozen 25% glycerol (−80°C) cell stocks in 14-mL round-bottom Falcon tubes containing 2-mL LB and cultured at 37°C with shaking (250 rpm) for 24 h. At *t* = 24 h, stationary-phase cells were diluted in PBS to achieve the desired cell density (∼10^6^-10^7^ cells/mL) and analyzed with a flow cytometer. Cells were excited at 488 nm wavelength and the green fluorescence signals were measured with the 530/30 nm bandpass filter. Of note, we focused on stationary-phase cells because the stringent response is significantly upregulated upon carbon and nitrogen source depletion (10, 14, 35). The selected promoters, including P*_talA_*, P*_rpoE_*, P*_yohC_*, P*_osmE_*, P*_rssB_*, P*_wrbA_*, P*_rpsA_*, P*_dksA_*, P*_rpsT_*, P*_pcnB_*, P*_rluC_*, and P*_yajC_* were obtained from the promoter strain collection (28).

### PI and SYTOX green staining.

Cells before and after antibiotic treatment were collected and diluted in 0.85% sodium chloride solution and then stained with 20 μM PI or 5 μM SYTOX Green to assess membrane permeability. PI is a red fluorescent dye and SYTOX Green is a green fluorescent dye. Both chemicals can only penetrate cells whose membrane integrity is compromised. Upon binding to DNA and RNA, the dye emits fluorescence. After adding the dye, cells were incubated for 15 min at 37°C in the dark. Stained cells were analyzed with a flow cytometer. Cells whose membranes were compromised by treating with 70% ethanol for 1 h served as a positive control; stained live cells were used as a negative control (Fig. S4). Forward scatter and side scatter parameters of unstained live cells were used to gate the cells on the flow-cytometry diagram. Cells were excited at a wavelength of 488 nm for green fluorescence and 561 nm for red fluorescence. A 615/20 nm bandpass filter was used for red fluorescence. A 530/30 nm bandpass filter was used for green fluorescence.

### Ciprofloxacin uptake assay.

Overnight cultures of E. coli MG1655 WT and Δ*waaG* were diluted 100-fold in 14-mL Falcon tubes containing 2 mL LB media and cultured at 37°C with shaking (250 rpm). Once the cultures reached the exponential phase (*t* = 3h, OD_600_∼0.5), cells were treated with ciprofloxacin (3 μg/mL) for 5 h. Cultures left untreated served as negative controls. Of note, cell numbers were quantified with flow cytometry before and after ciprofloxacin treatment. PI staining verified that ciprofloxacin does not significantly permeabilize the cells (Fig. S10A). After ciprofloxacin treatment, cells were washed twice with ice-chilled PBS (1X) solution by centrifugation (at 13,300 rpm for 3 min) at 4°C to remove the antibiotic from the supernatant (52, 53). After the final washing step, the resulted cell pellets were immediately resuspended in lysis buffer (0.1 M glycine-HCl at pH∼3.0) (52, 53), and the volume of the buffer was adjusted to obtain a cell density of 10^8^ cells/mL. Then, the cells were incubated at room temperature for 2 h until they were completely lysed by the buffer solution, which was verified by PI staining (Fig. S10B) (52, 53). The supernatants, to which the intracellular ciprofloxacin was released, were collected by centrifugation (at 13,300 rpm for 3 min) and transferred to the wells of a flat-bottom 96-well plate (300 μl supernatant per well) to measure the ciprofloxacin concentration by a plate reader. The excitation and emission wavelengths were 275 nm and 410 nm, respectively (52, 53). The same procedure was applied to the negative controls to measure the background fluorescence. Calibration curves were generated by diluting ciprofloxacin in the supernatant of the lysed cells obtained from the negative controls (Fig. S10C) (52, 53).

### Quantifying nongrowing cells.

E. coli MG1655 MO and Δ*waaG* cells harboring the IPTG-inducible *mCherry* expression cassette were grown in overnight precultures with 1 mM IPTG and 50 μg/mL kanamycin. Some cells were treated with CPZ (0.25 mM) at OD_600_=1.0 before their transition to the stationary phase. At the late stationary phase (*t* = 24 h), the cells were washed with PBS to remove the IPTG, diluted 100-fold in 2 mL LB in 14-mL Falcon tubes, and grown in the incubator with shaking and without IPTG. At the indicated times before and after antibiotic treatment, cells were collected and diluted in PBS to a cell density of 10^6^–10^7^ cells/mL. Diluted cells were analyzed by flow cytometry to measure mCherry. Forward scatter and side scatter parameters were used to gate the live-cell subpopulations, cell debris and instrumental noise on the flow-cytometry diagram, as described previously (83). Cells were excited at a wavelength of 561 nm and red fluorescence signals were collected by a 615/20 nm bandpass filter.

### DiSC_3_(5) assay.

The underlying mechanism of this phenomenon and the measurement of PMF components by DiSC_3_(5) have been well-documented elsewhere (65–67). E. coli MG1655 WT and Δ*waaG* cells at stationary phase (*t* = 24 h) were collected and washed twice with an assay buffer (5 mM HEPES and 20 mM glucose) (66). The cell density was adjusted to OD_600_=1.0 and cells were stained with 10 μM DiSC_3_(5) and left in the dark for 10 min to stabilize the dye fluorescence. Fluorescence was measured at designated times using a plate reader. The excitation and emission wavelengths were 620 nm and 670 nm, respectively. CPZ (0.25 mM) and polymyxin B (32 μg/mL) were added 30 min after reaching equilibrium to dissipate ΔpH and ΔΨ, respectively. Polymyxin B at 32 μg/mL was based on a previous study (66). The CPZ concentration of 0.25 mM was based on our previous study, which showed that this concentration decreases the stationary-phase cell metabolism and reduces persister formation (17).

### ATP measurement.

The intracellular ATP levels of the E. coli MG1655 WT and Δ*waaG* strains were measured at the indicated growth phase using a BacTiter-Glo Microbial Cell Viability assay kit (Promega Corporation, Catalog# G8230) according to the manufacturer’s instructions. A standard curve was generated using ATP solutions of known concentrations. LB broth was used to measure background luminescence with a plate reader.

### Biofilm assay.

As previously described (63), 10 μl of overnight precultures of E. coli WT and Δ*waaG* strains were inoculated onto sterile PES membranes (0.2 μm pore size, 25 mm diameter) atop LB agar plates and incubated for 24 h at 37°C. PES membranes were aseptically removed from the agar plates and vortexed (2000 rpm) in 4 mL sterile PBS for 1 min. Cells were harvested by centrifugation, resuspended in LB broth, and diluted in 2 mL LB medium to a cell density of ∼10^7^ cells/mL and treated with ofloxacin (5 μg/mL) for 5 h. Before antibiotic treatment, 10 μl cell suspensions were serially diluted in PBS to determine the initial number of cells. After the treatment, cells were collected, washed, and plated on an LB agar plate to determine the number of persisters.

### Statistical analysis.

High-throughput screening of the promoter library and the Keio collection was performed only once. At least three independent biological replicates were used for subsequent validation assays. Each data point in the figures represents the mean value ± standard deviation (SD). Statistical analyses were performed using GraphPad Prism 9.3.0 software. One-way analysis of variance (ANOVA) with Dunnett’s *post hoc* test or a two-tailed Student's *t* test with unequal variance were used to determine the statistical significance. Heatmaps were generated using a built-in function in GraphPad Prism 9.3.0. Bimodal curves were compared using a nonlinear model where F-statistics were used (17, 84). The *P* value threshold was selected as *, P < 0.05, **, P < 0.01, ***, P < 0.001, ****, P < 0.0001, and *ns* nonsignificant.
