# Supplementary material for: High-Throughput Screening of a Promoter Library Reveals New Persister Mechanisms in Escherichia Coli
Source: Microbiol Spectr. 2022 Feb 23;10(1):e02253-21. doi: 10.1128/spectrum.02253-21 (PMC8865558; doi:10.1128/spectrum.02253-21)
Supplement: SUPPLEMENTAL FILE 1 — Supplemental material. Download SPECTRUM02253-21_Supp_1_seq6.pdf, PDF file, 1.0 MB [file spectrum02253-21_supp_1_seq6.pdf]

Supplementary Information

High-Throughput Screening of a Promoter Library Reveals New Persister Mechanisms in *Escherichia Coli*

Sayed Golam Mohiuddin[1], Aslan Massahi[1] and Mehmet A. Orman[1]*

[1] Department of Chemical and Biomolecular Engineering, University of Houston, Houston, TX, USA

*Correspondence to: S222 Engineering Bldg 1, 4726 Calhoun Rd, Houston, TX 77204, Phone: 713-743-6785, Email: morman@central.uh.edu

Supplementary Figures

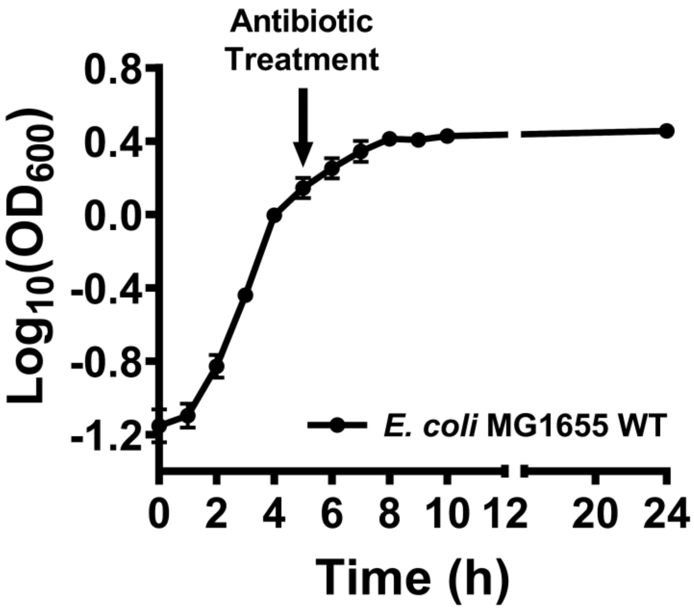

Fig. S1. The growth curve of *Escherichia coli* MG1655 WT cells. The growth of *E. coli* MG1655 WT cells in a 96-well plate. OD_{600} represents the optical density measured at a wavelength of 600 nm. N=4. Data points represent mean ± standard deviation (SD).

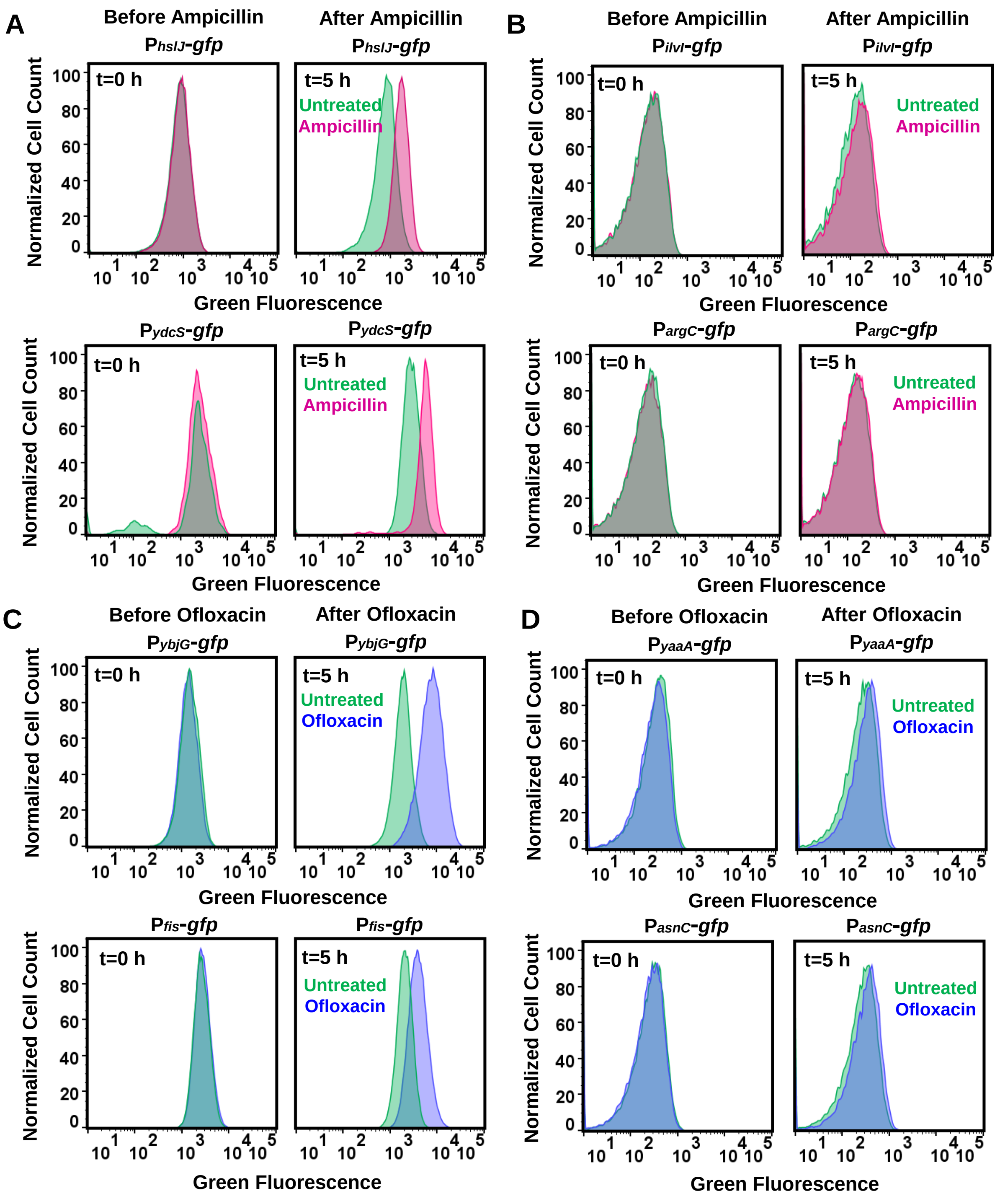

Fig. S2. Validation of high-throughput screening of the *E. coli* promoter library. GFP expression from the indicated promoter reporters was measured by flow cytometry in early stationary phase *E. coli* MG1655 cells after 5-h ampicillin (200 µg/ml) (**A-B**) and ofloxacin (5 µg/ml) (**C-D**) treatments. A representative replicate is shown, but similar data were obtained from all independent biological replicates. N=4.

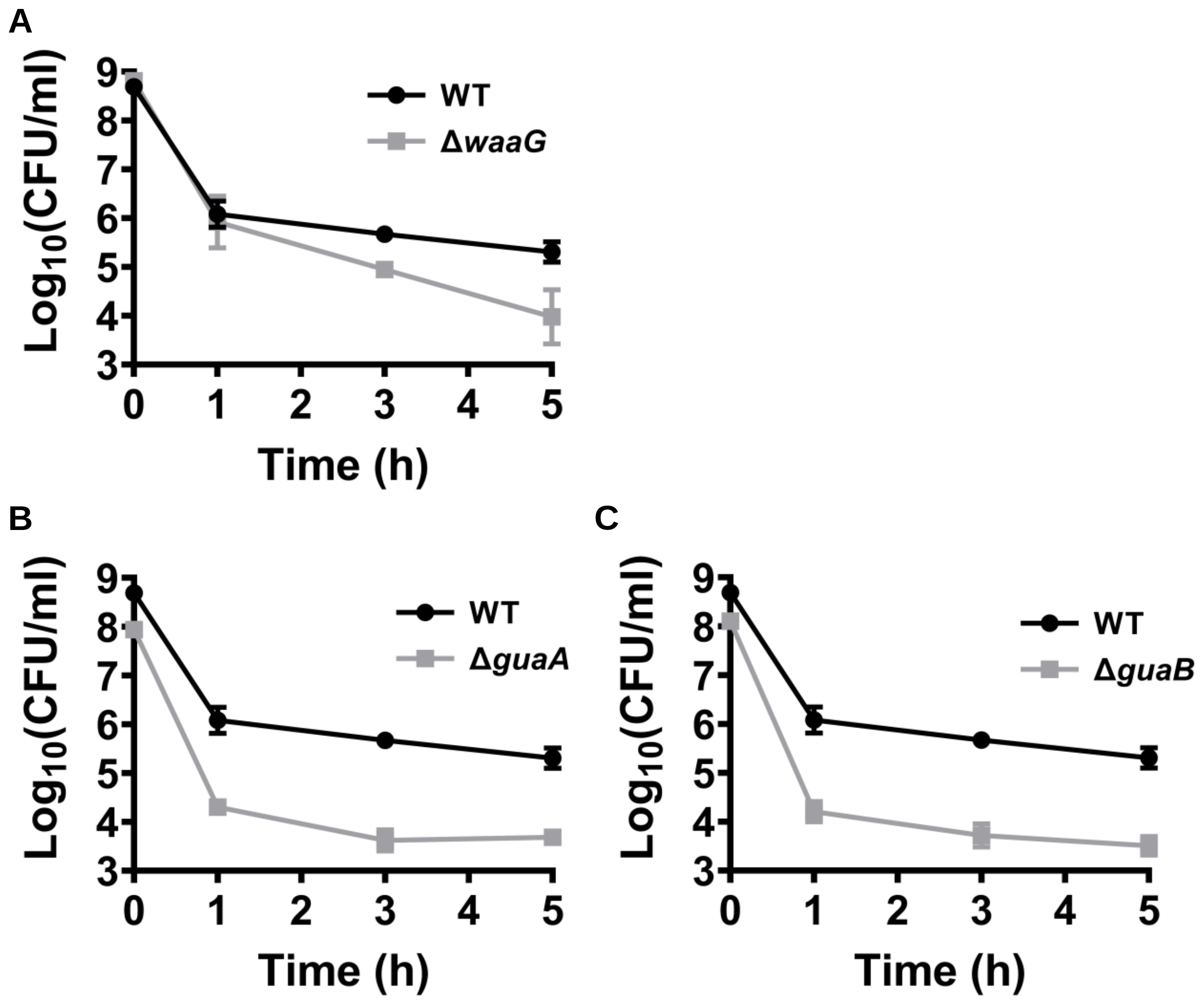

Fig. S3. Bi-phasic kill curves of identified mutants. (A-C) Persisters kill curves were generated by treating exponential phase cultures of *E. coli* MG1655 WT, Δ*waaG*, Δ*guaA*, and Δ*guaB* strains with ofloxacin for 5 h. At indicated time points, treated cells were collected to quantify CFU levels. N=4. Data points represent mean ± standard deviation (SD).

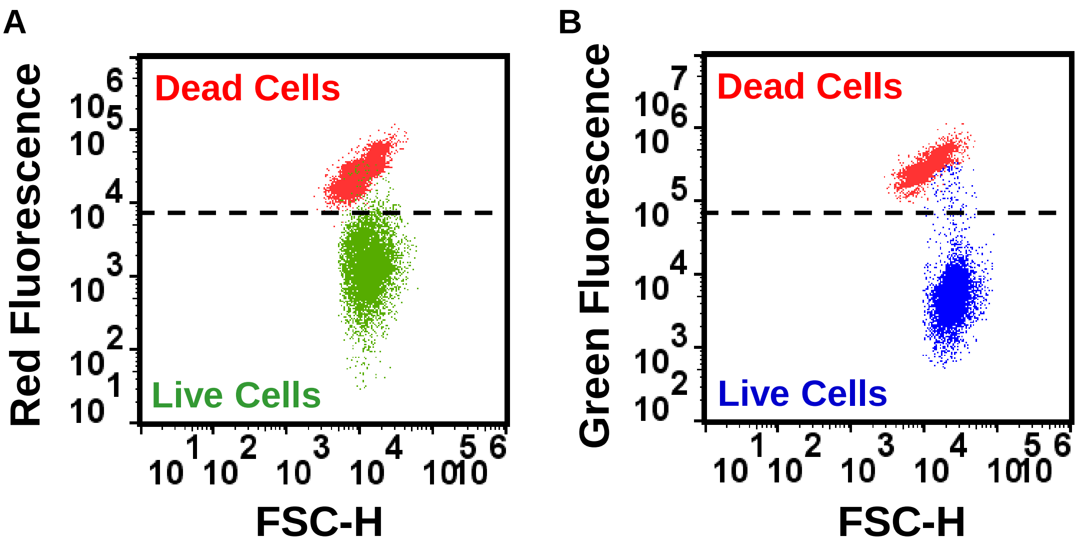

Fig. S4. PI and SYTOX green staining of live and dead cells. (A) Live and ethanol (70% v/v) treated dead cells were stained with PI and analyzed by flow cytometry to determine live and dead cells populations. A representative flow cytometry diagram is shown in the figure. Similar results were obtained from all independent biological replicates. N=4. **(B)** Live and ethanol (70% v/v) treated dead cells were stained with SYTOX green dye and analyzed by flow cytometry to determine live and dead cells populations. A representative flow cytometry diagram is shown in the figure. Similar results were obtained from all independent biological replicates. N=4. FSC: Forward Scatter.

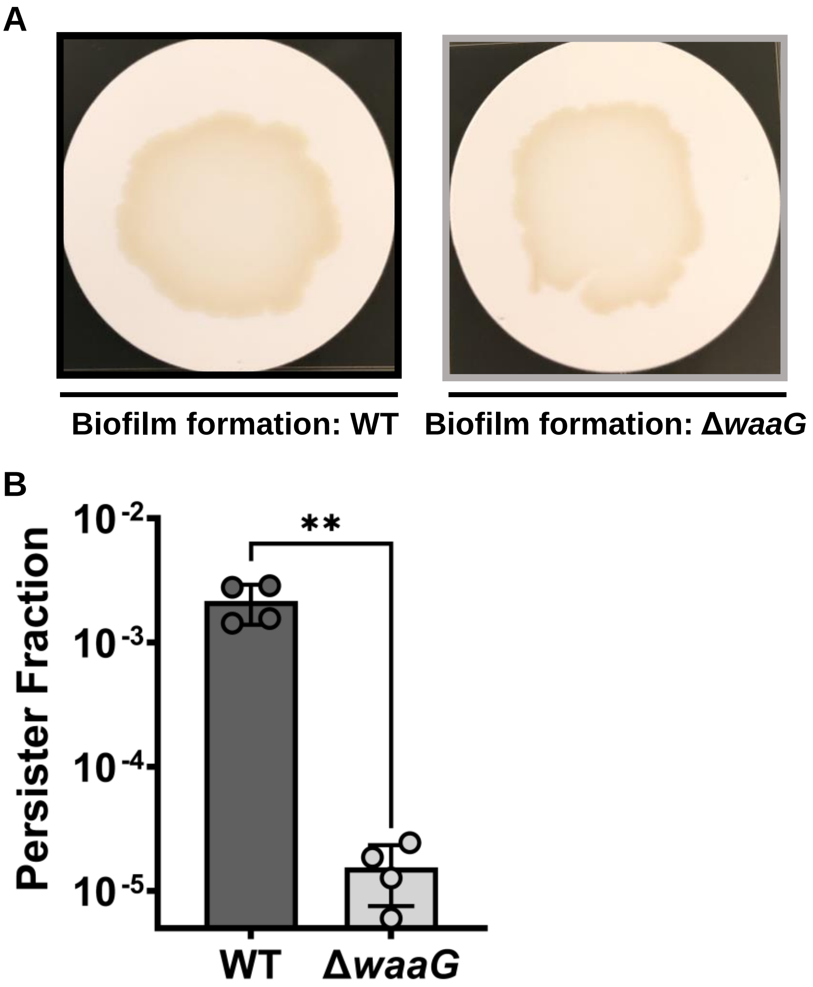

Fig. S5. Deletion of the *waaG* gene reduced persister levels in biofilms. (A) *E. coli* WT and Δ*waaG* biofilms on polyethersulfone membranes. **(B)** Cells from the biofilms were collected (see **Materials and Methods**), diluted in fresh medium, and treated with ofloxacin for 5 h to determine the persister levels N=4. For pairwise comparison, a two-tailed student t-test with unequal variance was used where **$P<0.01$. Data points represent mean ± standard deviation (SD).

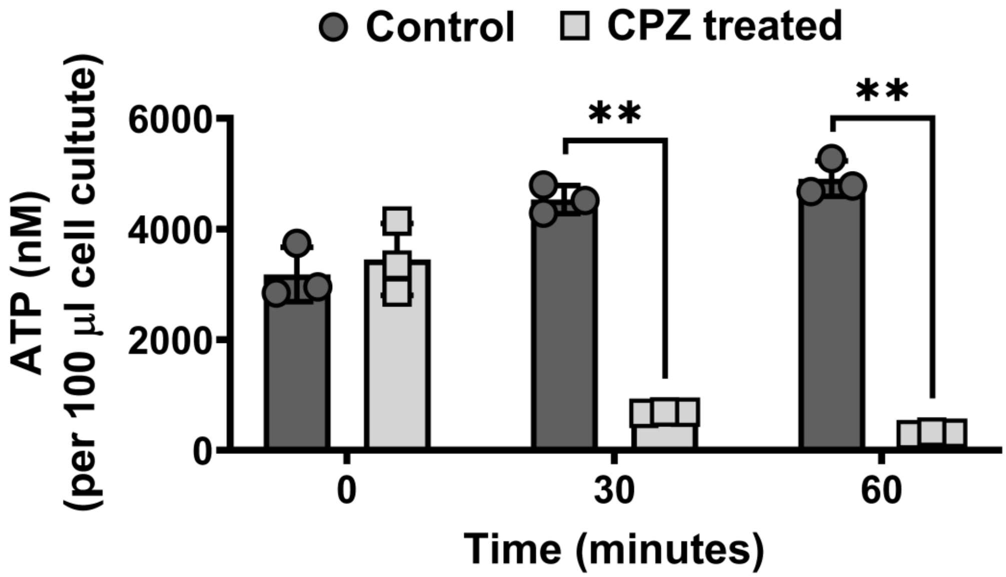

Fig. S6. ATP measurement of CPZ treated cells. Early stationary phase cells were treated with CPZ (0.25 mM) and ATP levels were measured using a BacTiter-Glo™ Microbial Cell Viability Assay kit at designated time points. The untreated culture served as control. N=3. For pairwise comparisons, one-way ANOVA with Dunnett's post hoc test was used where **P <0.01. Data points represent mean ± standard deviation (SD).

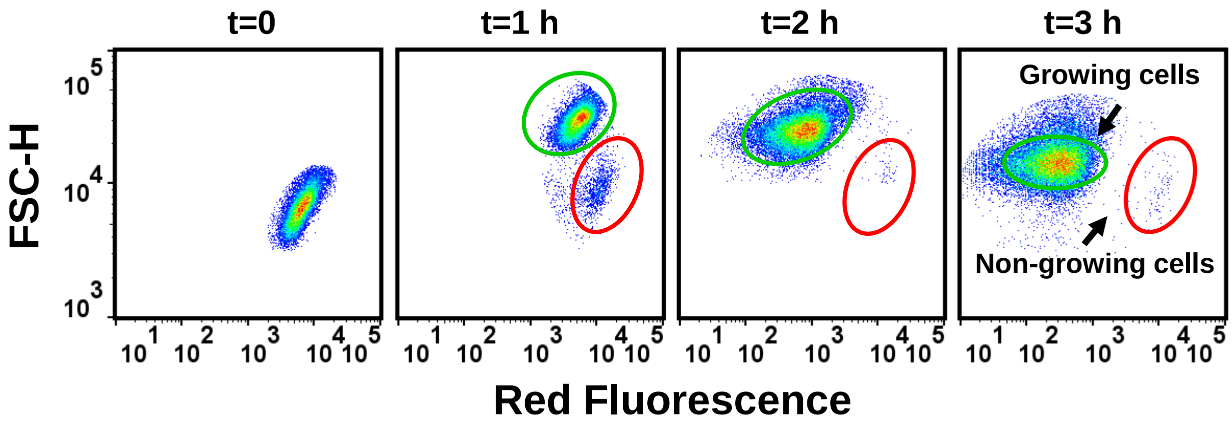

Fig. S7. Quantification of non-growing cells in CPZ pre-treated *E. coli* cultures. *E. coli* MG1655 MO cells expressing an IPTG-inducible *mCherry* were grown with IPTG in overnight pre-cultures. The cells were treated with 0.25 mM CPZ before they transitioned to the stationary phase (OD_{600}=1.0) in overnight pre-cultures. At the late-stationary phase (t = 24 h), overnight pre-cultures were diluted 100-fold in a medium without IPTG. At the indicated times, cells were collected and analyzed by flow cytometry. A representative replicate is shown, but similar data were obtained for all biological replicates. N=4.

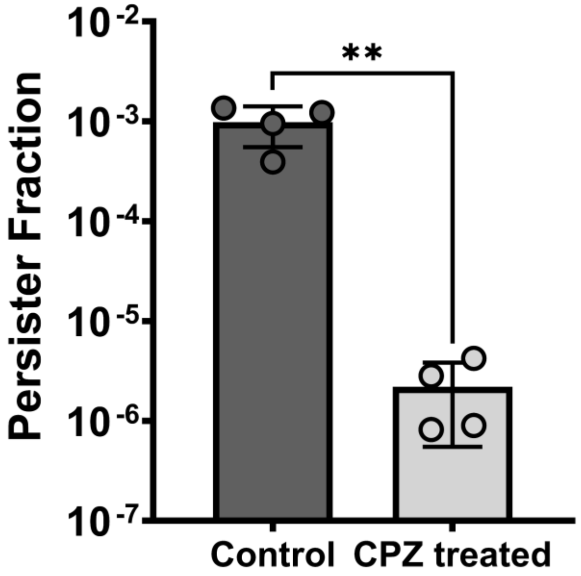

Fig. S8. Quantification of persister levels in CPZ pre-treated *E. coli* cells. *E. coli* MG1655 WT cells were treated with CPZ (0.25 mM) at OD_{600}=1.0 in the overnight pre-cultures. At the late stationary phase (t= 24 h), cells were collected, washed to remove CPZ, inoculated in fresh media, and treated with ofloxacin (5 µg/ml) for 5 h to determine the persister levels. N=4. For pairwise comparison, a two-tailed student t-test with unequal variance was used. **P<0.01. Data points represent mean ± standard deviation (SD).

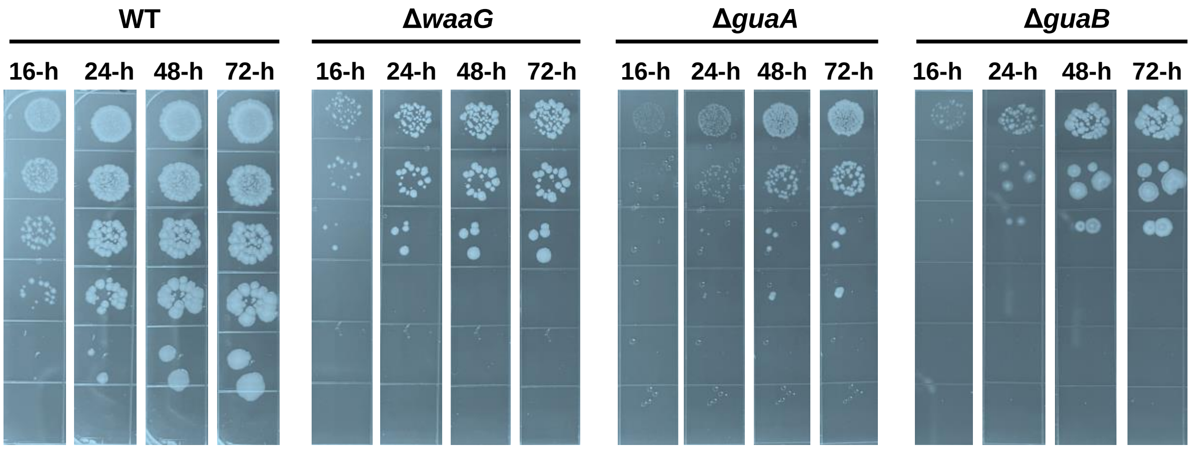

Fig. S9. Effects of longer incubations on persister recovery. Exponential phase *E. coli* MG1655 WT, Δ*waaG*, Δ*guaA*, and Δ*guaB* cells were treated with ofloxacin (5 µg/ml) for 5 h. After the treatment, cells were collected, washed, serially diluted, spotted on LB agar plates, and incubated for 72 h at 37 °C. Images of the plates were captured at indicated time points. A representative replicate is shown, but similar data were obtained for all biological replicates. N=4.

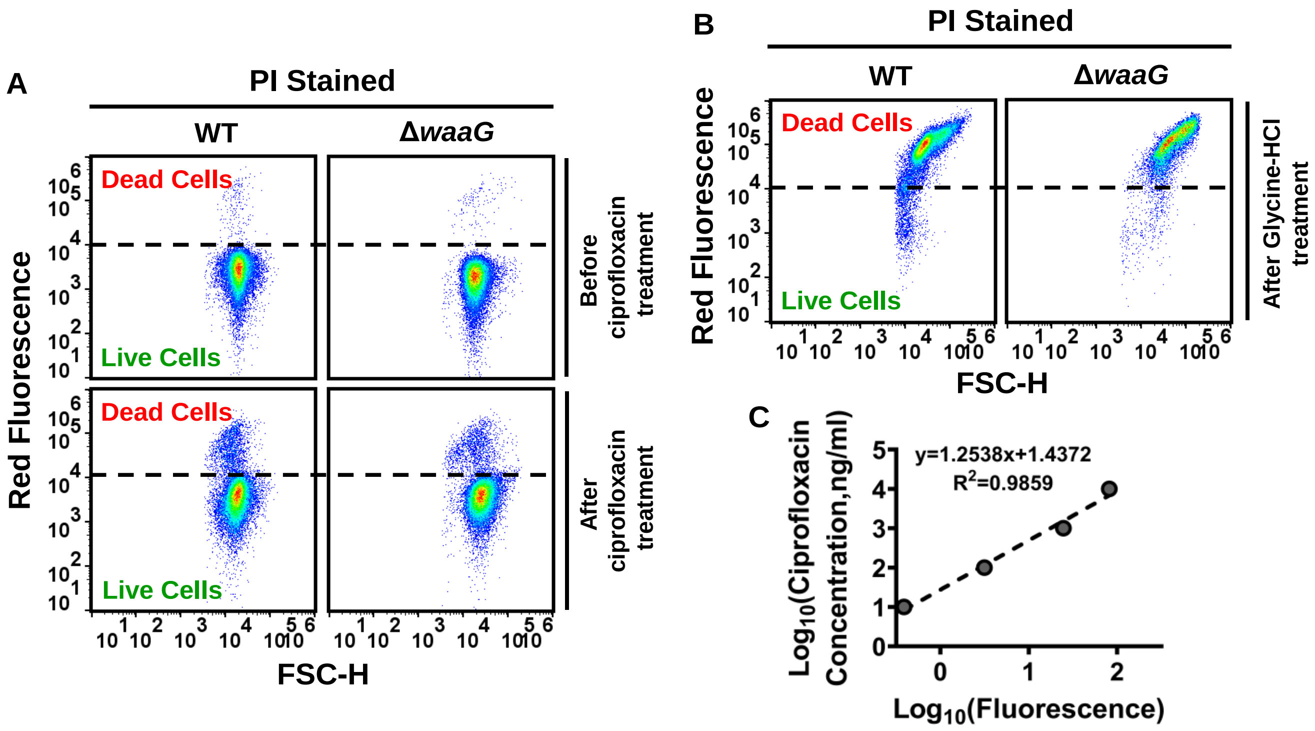

Fig. S10. Ciprofloxacin uptake assay. (A-B) Exponential phase *E. coli* MG1655 WT and Δ*waaG* cells (t~3 h, ~10^8 cells/ml) were treated with ciprofloxacin (3 µg/ml) for 5 h. Untreated and treated cells were collected, washed with PBS (1X), resuspended in glycine-HCl buffer (pH=3.0), and incubated for 2 h at room temperature. Cells at indicated time points were stained with propidium iodide (PI) and analyzed by flow cytometry to measure membrane permeability. Cells treated with 70% v/v ethanol were used as a positive control. PI stained live cells were used as a negative control. These controls were used to gate live and dead cells populations (**see Supp. Fig. S4**). A representative replicate is shown, but similar data were obtained for all biological replicates. N=4. **(C)** The calibration curve of ciprofloxacin uptake assay. Ciprofloxacin at known concentrations was diluted in the supernatants of untreated cell cultures lysed by the glycine-HCl buffer, and fluorescence levels of the solutions were measured with a plate reader. N=4.

Supplementary Tables

Table S1: Upregulated promoters and their corresponding genes in antibiotic-treated cultures. *Data obtained from EcoCyc* (1).

A. Ampicillin Treatment		
**Identified Promoters**	**Associated Gene(s)**	**Gene Function(s)**
P_{hslJ}	*hslJ*	Lipoprotein implicated in Novobiocin resistance. HslJ is a membrane-bound lipoprotein.
P_{spy}	*spy*	ATP-independent periplasmic chaperone. Spy is an ATP-independent periplasmic chaperone that prevents protein aggregation and that assists in protein refolding.
P_{ycfJ}	*ycfJ*	YcfJ has the similarity to Proteus mirabilis UmoD, which is involved in the flagellar synthesis, swarming, and cell elongation.
P_{yacH}	*yacH*	DUF3300 domain-containing protein YacH (potentially a membrane protein).
P_{ydeI}	*ydeI*	BOF family protein YdeI. YdeI is involved in the cellular response to hydrogen peroxide stress.
P_{nlpD}	*nlpD*	Murein hydrolase activator NlpD. NlpD is a divisome associated, outer membrane lipoprotein that activates the peptidoglycan (PG) hydrolase AmiC.
	rpoS	RNA polymerase, sigma S (sigma 38) factor.
P_{yhbU}	*ubiU*	Ubiquinone biosynthesis protein UbiU
	ubiV	Ubiquinone biosynthesis protein UbiV
P_{ygdI}	*ygdI*	DUF903 domain-containing lipoprotein YgdI
P_{yfcI}	*yfcI*	Recombination-promoting nuclease RpnB
P_{cpsG}	*wzxC*	Colanic acid repeat unit flippase. *wzxC* is located in a cluster of genes responsible for the production of the extracellular polysaccharide.
	wcaJ	UDP-glucose:undecaprenyl-phosphate glucose-1-phosphate transferase
	cpsG	Phosphomannomutase
	cpsB	Mannose-1-phosphate guanylyltransferase. CpsB is involved in the biosynthesis of the capsular polysaccharide colanic acid.
	wcaI	Colanic acid biosynthesis fucosyltransferase
	gmm	GDP-mannose mannosyl hydrolase
	fcl	GDP-L-fucose synthase
	gmd	GDP-mannose 4,6-dehydratase
	wcaF	Colanic acid biosynthesis acetyltransferase WcaF
	wcaE	WcaE is a fucosyltransferase that catalyzes the transfer of a fucosyl residue to the UPP-linked acetylated disaccharide during biosynthesis of the extracellular polysaccharide colanic acid.
	wcaD	Colanic acid polymerase. *wcaD* is located within a cluster of genes that are responsible for the production of the extracellular polysaccharide, colanic acid.
	wcaC	Colanic acid biosynthesis galactosyltransferase
P_{deoA}	*deoA*	Thymidine phosphorylase / uracil phosphorylase
P_{yiaT}	*yiaT*	Outer membrane protein YiaT
P_{galU}	*galU*	UTP—glucose-1-phosphate uridylyltransferase
P_{rcsA}	*rcsA*	DNA-binding transcriptional activator
P_{yidK}	*yidK*	putative transporter
	yidJ	YidJ is a putative Cys-type sulfatase.
P_{glnW}	*glnX*	tRNA-Gln(CUG)
	glnV	tRNA-Gln(CUG). tRNA(*glnV*) is one of four glutamine tRNAs.

	metU	tRNA-Met(CAU). tRNA(*metU*) is one of six methionine tRNAs, and one of two methionine tRNAs that operates during protein elongation rather than initiation of protein synthesis.
	glnW	tRNA-Gln(UUG). tRNA(*glnW*) is one of four glutamine tRNAs.
P_{panE}	*panE*	2-dehydropantoate 2-reductase
	yajL	Protein/nucleic acid deglycase 3
P_{arpA}	*arpA*	Regulator of acetyl CoA synthetase
P_{yncE}	*yncE*	PQQ-like domain-containing protein
P_{ypfG}	*ypfG*	DUF1176 domain-containing protein
P_{nagA}	*nagA*	N-acetylglucosamine-6-phosphate deacetylase
	nagB	Glucosamine-6-phosphate deaminase
	nagC	DNA-binding transcriptional dual regulator
P_{yeeJ}	*yeeJ*	Inverse autotransporter adhesin
P_{fecA}	*fecA*	Ferric citrate outer membrane transporter. FecA is an outer membrane (OM) protein that mediates the citrate dependent import of ferric iron.
	fecB	Ferric citrate ABC transporter periplasmic binding protein. *fecB* encodes the periplasmic substrate binding component of the iron dicitrate ABC transporter.
	fecC	Ferric citrate ABC transporter membrane subunit FecC. FecC is one of two (along with FecD) integral membrane proteins of the iron dicitrate ABC transport system.
	fecD	Ferric citrate ABC transporter membrane subunit FecD. FecD is one of two (along with FecC) integral membrane proteins of the iron dicitrate ABC transport system.
	fecE	Ferric citrate ABC transporter ATP binding subunit. *fecE* encodes a hydrophilic protein that is found in the membrane fraction.
P_{yeiT}	*preT*	NAD-dependent dihydropyrimidine dehydrogenase subunit PreT
	preA	NAD-dependent dihydropyrimidine dehydrogenase subunit PreA
P_{yeiP}	*yeiP*	Elongation factor P-like protein
P_{b1998}	*yoeA*	CP4-44 prophage; TonB-dependent receptor plug domain-containing protein
P_{emrR}	*emrR*	DNA-binding transcriptional repressor MprA
	emrA	Multidrug efflux pump membrane fusion protein EmrA
	emrB	Multidrug efflux pump membrane subunit EmrB
P_{ygcB}	*ygcB*	CRISPR-associated endonuclease/helicase Cas3

**B. Ofloxacin Treatment**		
**Identified Promoters**	**Associated Gene(s)**	**Gene Function(s)**
P_{recN}	*recN*	DNA repair protein RecN. RecN functions in the recombinational repair of DNA double-strand breaks
P_{recA}	*recA*	DNA recombination/repair protein RecA. RecA is a DNA strand exchange protein of homologous recombination.
	recX	RecA inhibitor RecX. RecX interacts directly with RecA, inhibits its ssDNA-dependent ATPase.
P_{ybjG}	*ybjG*	Undecaprenyl pyrophosphate phosphatase. YbjG is an inner membrane protein with four transmembrane domains.
P_{lexA}	*lexA*	DNA-binding transcriptional repressor LexA
	dinF	DNA damage-inducible protein F. DinF is a member of the Multi Antimicrobial Extrusion (MATE) Family transporters.
P_{sulA}	*sulA*	Cell division inhibitor SulA. SulA inhibits cell division during SOS response.
P_{htrL}	*htrL*	Protein HtrL

P_{yjfI}	*yjfI*	DUF2170 domain-containing protein YjfI
	rlmB	23S rRNA 2'-O-ribose G2251 methyltransferase. RlmB is the methyltransferase responsible for methylation of 23S rRNA at the 2'-O position of the ribose at the G2251 nucleotide.
	yjfJ	PspA family protein YjfJ. YjfJ belongs to the PspA/IM30 family of proteins.
	nsrR	DNA-binding transcriptional dual regulator NsrR
	rnr	RNase R. RNase R is a ribonuclease that has been implicated in rRNA maturation, mRNA degradation during the stationary phase, degradation of polyadenylated mRNAs, and tmRNA-mediated degradation of non-stop mRNAs.
P_{dinB}	*dinB*	DNA polymerase IV. *dinB* encodes DNA polymerase IV (DNA pol IV, pol IV), which belongs to the Y family of specialized DNA polymerases. Pol IV is part of a double strand break (DSB)-repair-dependent stress-induced mutagenesis (SIM) pathway.
	yafN	Antitoxin YafN. YafN is the antitoxin for the ribosome-associated mRNA interferase toxin YafO.
	yafO	Ribosome-dependent mRNA interferase toxin YafO. YafO is the toxin of the YafO-YafN toxin-antitoxin system, acting as a ribosome-dependent mRNA interferase.
	yafP	Putative acyltransferase with acyl-CoA N-acyltransferase domain
P_{rfbB}	*rfbA*	Glucose-1-phosphate thymidylyltransferase 1
	rfbB	dTDP-glucose 4,6-dehydratase 1
	rfbC	dTDP-4-dehydrorhamnose 3,5-epimerase
	rfbD	dTDP-4-dehydrorhamnose reductase
	rfbX	Polyisoprenol-linked O16-antigen repeat unit flippase. RfbX, also known as Wzx and WzxB, is the flippase that translocates undecaprenylpyrophosphate (Und-PP) linked O-antigen subunits across the inner membrane.
P_{pyrG}	*pyrG*	CTP synthetase. CTP synthetase catalyzes the glutamine- or ammonia-dependent synthesis of CTP from UTP, the final step in the *de novo* biosynthesis of CTP.
P_{ycbZ}	*ycbZ*	Putative ATP-dependent protease YcbZ
P_{sthA}	*sthA*	Soluble pyridine nucleotide transhydrogenase
P_{ylaC}	*ylaC*	Putative inner membrane protein. YlaC is an inner membrane protein with two predicted transmembrane domains.
P_{lpd}	*lpd*	Lipoamide dehydrogenase
P_{ileX}	*ileX*	tRNA-Ile(CAU). tRNA(*ileX*) is one of five isoleucine tRNAs.
P_{glyU}	*glyU*	tRNA-Gly(CCC). tRNA(*glyU*) is one of six glycine tRNAs.
P_{lpxC}	*lpxC*	UDP-3-O-acyl-N-acetylglucosamine deacetylase. LpxC catalyzes the second reaction and the first committed step in lipid A biosynthesis.
P_{rpsU}	*rpsU*	30S ribosomal subunit protein S21.
	dnaG	DNA primase. DNA primase catalyzes the synthesis of RNA primers on single-stranded template DNA.
P_{greA}	*greA*	Transcription elongation factor GreA
P_{yeaZ}	*yeaZ*	N^6-L-threonylcarbamoyladenine synthase, TsaB subunit
P_{ychF}	*ychF*	Redox-responsive ATPase YchF
P_{sspA}	*sspA*	Stringent starvation protein A
	sspB	ClpXP protease specificity-enhancing factor
P_{gyrB}	*gyrB*	DNA gyrase subunit B. The GyrB subunit of DNA gyrase is required for the ATPase activity of the enzyme.
P_{yfcE}	*yfcE*	Phosphodiesterase YfcE.
P_{polB}	*polB*	DNA polymerase II. Pol II catalyzes both a DNA polymerase activity and a 3' to 5' exonuclease activity.

P_{yebO}	*yebO*	Uncharacterized protein YebO.
P_{trxA}	*trxA*	thioredoxin 1. Thioredoxins are small electron transfer proteins that contain a cysteine disulfide/dithiol active site with the amino acid sequence motif Cys-X-X-Cys
P_{rfaQ}	*waaQ*	Lipopolysaccharide core heptosyltransferase 3
	waaG	Lipopolysaccharide glucosyltransferase I
	waaP	Lipopolysaccharide core heptose (I) kinase
	waaS	Lipopolysaccharide core biosynthesis protein WaaS
	waaB	UDP-D-galactose:(glucosyl)lipopolysaccharide-1,6-D-galactosyltransferase
	waaO	UDP-D-glucose:(glucosyl)LPS α-1,3-glucosyltransferase
	waaJ	UDP-glucose:(glucosyl)LPS α-1,2-glucosyltransferase
P_{rpmE}	*rpmE*	50S ribosomal subunit protein L31
P_{b0501}	*ybbD*	Putative uncharacterized protein YbbD
P_{sbmC}	*sbmC*	DNA gyrase inhibitor
P_{yfgB}	*yfgB*	23S rRNA m2A2503 methyltransferase/tRNA m2A37 methyltransferase
P_{yafL}	*yafL*	NlpC/P60 family protein YafL
P_{guaB}	*guaB*	Inosine 5'-monophosphate dehydrogenase
	guaA	GMP synthetase. GMP synthetase catalyzes the glutamine- or ammonia-dependent synthesis of GMP from XMP
P_{insA_7}	*insA_7*	KpLE2 phage-like element; IS1 repressor protein InsA
P_{mltD}	*mltD*	Membrane-bound lytic murein transglycosylase D
P_{ahpF}	*ahpF*	Alkyl hydroperoxide reductase, AhpF component
	ahpC	Alkyl hydroperoxide reductase, AhpC component. AhpC is the peroxidase component of alkyl hydroperoxide reductase belonging to the family of typical 2-Cys peroxiredoxins with two conserved redox-active cysteines.
P_{rpmB}	*rpmB*	50S ribosomal subunit protein L28. The L28 protein is a component of the 50S subunit of the ribosome and is required for ribosome assembly.
	rpmG	50S ribosomal subunit protein L33. The L33 protein is a component of the 50S subunit of the ribosome.
P_{dusB}	*dusB*	tRNA-dihydrouridine synthase B.
	fis	DNA-binding transcriptional dual regulator Fis.

Table S2: Single mutant strains and oligonucleotides used in this study.

A. Mutant Strains (Keio collection)	Source
Escherichia coli K-12 BW25113 Δ*nagA*	Keio collection, Cat# OEC4988
Escherichia coli K-12 BW25113 Δ*wcaI*	Keio collection, Cat# OEC4988
Escherichia coli K-12 BW25113 Δ*cpsG*	Keio collection, Cat# OEC4988
Escherichia coli K-12 BW25113 Δ*wzxC*	Keio collection, Cat# OEC4988
Escherichia coli K-12 BW25113 Δ*yfcI*	Keio collection, Cat# OEC4988
Escherichia coli K-12 BW25113 Δ*ydeI*	Keio collection, Cat# OEC4988
Escherichia coli K-12 BW25113 Δ*ypfG*	Keio collection, Cat# OEC4988
Escherichia coli K-12 BW25113 Δ*hslJ*	Keio collection, Cat# OEC4988
Escherichia coli K-12 BW25113 Δ*fecD*	Keio collection, Cat# OEC4988
Escherichia coli K-12 BW25113 Δ*wcaJ*	Keio collection, Cat# OEC4988
Escherichia coli K-12 BW25113 Δ*spy*	Keio collection, Cat# OEC4988
Escherichia coli K-12 BW25113 Δ*wcaD*	Keio collection, Cat# OEC4988
Escherichia coli K-12 BW25113 Δ*yncE*	Keio collection, Cat# OEC4988
Escherichia coli K-12 BW25113 Δ*wcaF*	Keio collection, Cat# OEC4988
Escherichia coli K-12 BW25113 Δ*galU*	Keio collection, Cat# OEC4988
Escherichia coli K-12 BW25113 Δ*cpsB*	Keio collection, Cat# OEC4988
Escherichia coli K-12 BW25113 Δ*rcsA*	Keio collection, Cat# OEC4988
Escherichia coli K-12 BW25113 Δ*fcl*	Keio collection, Cat# OEC4988

Escherichia coli K-12 BW25113 Δ*fecC*	Keio collection, Cat# OEC4988
Escherichia coli K-12 BW25113 Δ*fecA*	Keio collection, Cat# OEC4988
Escherichia coli K-12 BW25113 Δ*emrA*	Keio collection, Cat# OEC4988
Escherichia coli K-12 BW25113 Δ*yajL*	Keio collection, Cat# OEC4988
Escherichia coli K-12 BW25113 Δ*gmd*	Keio collection, Cat# OEC4988
Escherichia coli K-12 BW25113 Δ*nlpD*	Keio collection, Cat# OEC4988
Escherichia coli K-12 BW25113 Δ*yidK*	Keio collection, Cat# OEC4988
Escherichia coli K-12 BW25113 Δ*nagC*	Keio collection, Cat# OEC4988
Escherichia coli K-12 BW25113 Δ*preT*	Keio collection, Cat# OEC4988
Escherichia coli K-12 BW25113 Δ*wcaE*	Keio collection, Cat# OEC4988
Escherichia coli K-12 BW25113 Δ*emrB*	Keio collection, Cat# OEC4988
Escherichia coli K-12 BW25113 Δ*fecB*	Keio collection, Cat# OEC4988
Escherichia coli K-12 BW25113 Δ*arpA*	Keio collection, Cat# OEC4988
Escherichia coli K-12 BW25113 Δ*preA*	Keio collection, Cat# OEC4988
Escherichia coli K-12 BW25113 Δ*yacH*	Keio collection, Cat# OEC4988
Escherichia coli K-12 BW25113 Δ*yidJ*	Keio collection, Cat# OEC4988
Escherichia coli K-12 BW25113 Δ*yeiP*	Keio collection, Cat# OEC4988
Escherichia coli K-12 BW25113 Δ*ygcB*	Keio collection, Cat# OEC4988
Escherichia coli K-12 BW25113 Δ*emrR*	Keio collection, Cat# OEC4988
Escherichia coli K-12 BW25113 Δ*nagB*	Keio collection, Cat# OEC4988
Escherichia coli K-12 BW25113 Δ*fecE*	Keio collection, Cat# OEC4988
Escherichia coli K-12 BW25113 Δ*ygdI*	Keio collection, Cat# OEC4988
Escherichia coli K-12 BW25113 Δ*gmm*	Keio collection, Cat# OEC4988
Escherichia coli K-12 BW25113 Δ*ycfJ*	Keio collection, Cat# OEC4988
Escherichia coli K-12 BW25113 Δ*yeeJ*	Keio collection, Cat# OEC4988
Escherichia coli K-12 BW25113 Δ*wcaC*	Keio collection, Cat# OEC4988
Escherichia coli K-12 BW25113 Δ*yiaT*	Keio collection, Cat# OEC4988
Escherichia coli K-12 BW25113 Δ*panE*	Keio collection, Cat# OEC4988
Escherichia coli K-12 BW25113 Δ*deoA*	Keio collection, Cat# OEC4988
Escherichia coli K-12 BW25113 Δ*yoeA*	Keio collection, Cat# OEC4988
Escherichia coli K-12 BW25113 Δ*ubiU*	Keio collection, Cat# OEC4988
Escherichia coli K-12 BW25113 Δ*rpoS*	Keio collection, Cat# OEC4988
Escherichia coli K-12 BW25113 Δ*recA*	Keio collection, Cat# OEC4988
Escherichia coli K-12 BW25113 Δ*guaB*	Keio collection, Cat# OEC4988
Escherichia coli K-12 BW25113 Δ*waaG*	Keio collection, Cat# OEC4988
Escherichia coli K-12 BW25113 Δ*rpmG*	Keio collection, Cat# OEC4988
Escherichia coli K-12 BW25113 Δ*mltD*	Keio collection, Cat# OEC4988
Escherichia coli K-12 BW25113 Δ*recN*	Keio collection, Cat# OEC4988
Escherichia coli K-12 BW25113 Δ*fis*	Keio collection, Cat# OEC4988
Escherichia coli K-12 BW25113 Δ*yafO*	Keio collection, Cat# OEC4988
Escherichia coli K-12 BW25113 Δ*guaA*	Keio collection, Cat# OEC4988
Escherichia coli K-12 BW25113 Δ*waaO*	Keio collection, Cat# OEC4988
Escherichia coli K-12 BW25113 Δ*rfbD*	Keio collection, Cat# OEC4988
Escherichia coli K-12 BW25113 Δ*waaP*	Keio collection, Cat# OEC4988
Escherichia coli K-12 BW25113 Δ*dinB*	Keio collection, Cat# OEC4988
Escherichia coli K-12 BW25113 Δ*yebO*	Keio collection, Cat# OEC4988
Escherichia coli K-12 BW25113 Δ*yafL*	Keio collection, Cat# OEC4988
Escherichia coli K-12 BW25113 Δ*rlmB*	Keio collection, Cat# OEC4988
Escherichia coli K-12 BW25113 Δ*dusB*	Keio collection, Cat# OEC4988
Escherichia coli K-12 BW25113 Δ*yafP*	Keio collection, Cat# OEC4988
Escherichia coli K-12 BW25113 Δ*dinF*	Keio collection, Cat# OEC4988
Escherichia coli K-12 BW25113 Δ*waaQ*	Keio collection, Cat# OEC4988
Escherichia coli K-12 BW25113 Δ*dnaG*	Keio collection, Cat# OEC4988

Escherichia coli K-12 BW25113 Δ*ycaQ*	Keio collection, Cat# OEC4988
Escherichia coli K-12 BW25113 Δ*sbmC*	Keio collection, Cat# OEC4988
Escherichia coli K-12 BW25113 Δ*polB*	Keio collection, Cat# OEC4988
Escherichia coli K-12 BW25113 Δ*sspB*	Keio collection, Cat# OEC4988
Escherichia coli K-12 BW25113 Δ*yfcE*	Keio collection, Cat# OEC4988
Escherichia coli K-12 BW25113 Δ*sspA*	Keio collection, Cat# OEC4988
Escherichia coli K-12 BW25113 Δ*yjfJ*	Keio collection, Cat# OEC4988
Escherichia coli K-12 BW25113 Δ*lpd*	Keio collection, Cat# OEC4988
Escherichia coli K-12 BW25113 Δ*sulA*	Keio collection, Cat# OEC4988
Escherichia coli K-12 BW25113 Δ*trxA*	Keio collection, Cat# OEC4988
Escherichia coli K-12 BW25113 Δ*rpmE*	Keio collection, Cat# OEC4988
Escherichia coli K-12 BW25113 Δ*yafN*	Keio collection, Cat# OEC4988
Escherichia coli K-12 BW25113 Δ*ychF*	Keio collection, Cat# OEC4988
Escherichia coli K-12 BW25113 Δ*ahpC*	Keio collection, Cat# OEC4988
Escherichia coli K-12 BW25113 Δ*htrL*	Keio collection, Cat# OEC4988
Escherichia coli K-12 BW25113 Δ*ahpF*	Keio collection, Cat# OEC4988
Escherichia coli K-12 BW25113 Δ*yjfI*	Keio collection, Cat# OEC4988
Escherichia coli K-12 BW25113 Δ*ylaC*	Keio collection, Cat# OEC4988
Escherichia coli K-12 BW25113 Δ*ycbZ*	Keio collection, Cat# OEC4988
Escherichia coli K-12 BW25113 Δ*ycaI*	Keio collection, Cat# OEC4988
Escherichia coli K-12 BW25113 Δ*recX*	Keio collection, Cat# OEC4988
Escherichia coli K-12 BW25113 Δ*waaS*	Keio collection, Cat# OEC4988
Escherichia coli K-12 BW25113 Δ*rnr*	Keio collection, Cat# OEC4988
Escherichia coli K-12 BW25113 Δ*greA*	Keio collection, Cat# OEC4988
Escherichia coli K-12 BW25113 Δ*yfgB*	Keio collection, Cat# OEC4988
Escherichia coli K-12 BW25113 Δ*rfbB*	Keio collection, Cat# OEC4988
Escherichia coli K-12 BW25113 Δ*rfbC*	Keio collection, Cat# OEC4988
Escherichia coli K-12 BW25113 Δ*rfbA*	Keio collection, Cat# OEC4988
Escherichia coli K-12 BW25113 Δ*ybjG*	Keio collection, Cat# OEC4988
Escherichia coli K-12 BW25113 Δ*ybbD*	Keio collection, Cat# OEC4988
Escherichia coli K-12 BW25113 Δ*rfbX*	Keio collection, Cat# OEC4988
Escherichia coli K-12 BW25113 Δ*waaB*	Keio collection, Cat# OEC4988
Escherichia coli K-12 BW25113 Δ*waaJ*	Keio collection, Cat# OEC4988
Escherichia coli K-12 BW25113 Δ*sthA*	Keio collection, Cat# OEC4988

**B. Bacterial Strains (*E. coli* MG1655)**	**Source or References**
Escherichia coli K-12 MG1655 Wild Type	(2)
Escherichia coli K-12 MG1655 MO Strain	(2)
Escherichia coli K-12 BW25113 Wild Type	Keio collection, Cat# OEC4988
MO Δ*waaG*	This study
Escherichia coli K-12 MG1655 Δ*nagA*	This study
Escherichia coli K-12 MG1655 Δ*wcaI*	This study
Escherichia coli K-12 MG1655 Δ*cpsG*	This study
Escherichia coli K-12 MG1655 Δ*wzxC*	This study
Escherichia coli K-12 MG1655 Δ*yfcI*	This study
Escherichia coli K-12 MG1655 Δ*guaB*	This study
Escherichia coli K-12 MG1655 Δ*waaG*	This study
Escherichia coli K-12 MG1655 Δ*rpmG*	This study
Escherichia coli K-12 MG1655 Δ*mltD*	This study
Escherichia coli K-12 MG1655 Δ*fis*	This study
Escherichia coli K-12 MG1655 Δ*yafO*	This study
Escherichia coli K-12 MG1655 Δ*guaA*	This study

Escherichia coli K-12 MG1655 Δ*waaO*	This study
**Plasmids**	**Source**
pMSs201	(3)

**C. Oligonucleotides to Generate Gene Deletions**			
**Mutation**	**Forward Primer (5' to 3')**	**Reverse Primer (5' to 3')**	**Source**
Δ*nagA*::KAN^R	TCCTTGCTCAGGGCAATATTT TTTAAAATCGGGGGTCAGAG TGTAGGCTGGAGCTGCTTC	TTACCTATCTGAGCTTGTCC GCCTGGTGTCATACTTTCTC TTAACGGCTGACATGGGAAT	Integrated DNA Technologies, Inc.
Δ*wcaI*::KAN^R	CCTATTTTCTCGCTGAGAAG CGTACCGGAGTACCCGGAT TGTGTAGGCTGGAGCTGCTTC	CCTGCCATCACAACTGGATA GAGTTTCGACTGCGCCATAA TTAACGGCTGACATGGGAAT	Integrated DNA Technologies, Inc.
Δ*cpsG*::KAN^R	TTCGGTCAGGGCCAACTATT GCCTGAAAAAGGGTAACGAT GTGTAGGCTGGAGCTGCTTC	TTAGCCCCTTACCCGCAGTG GGGTAAGGGAAGATCCGACA TTAACGGCTGACATGGGAAT	Integrated DNA Technologies, Inc.
Δ*wzxC*::KAN^R	ACGGTGTTCAAAGGTTTCGT TAACAAAGCGGCATATTGAT GTGTAGGCTGGAGCTGCTTC	GCCGCATCCGGCAACCGTTG TCGGAACCGAAAACAGCAACT TAACGGCTGACATGGGAAT	Integrated DNA Technologies, Inc.
Δ*yfcI*::KAN^R	ATTGTCGCGTCATCTTAAAT TTGAGGGGTGACGGAACGC CGTGTAGGCTGGAGCTGCTTC	TACAGCAAACATCCGCCAGTT AACTCCCGGTGTTACAGGATT AACGGCTGACATGGGAAT	Integrated DNA Technologies, Inc.
Δ*guaB*::KAN^R	GCGGCAATATTTATTAACCAC TCTGGTCGAGATATTGCCCGT GTAGGCTGGAGCTGCTTC	GATAATATAAATCGCCCGACA TGAAGTCGGGCGAAGAGAATT AACGGCTGACATGGGAAT	Integrated DNA Technologies, Inc.
Δ*waaG*::KAN^R	TGCCAGAAGATGCCCCTTCA GCTGACAGGAATGCACAATT GTGTAGGCTGGAGCTGCTTC	ATCTTTACCACGCCAAAGTGT GGCAAGCGGCTCTTTTAATTT AACGGCTGACATGGGAAT	Integrated DNA Technologies, Inc.
Δ*rpmG*::KAN^R	CCCGTGGCGAAAAGTACTAA GTACTTAGAGGAAATAAATCG TGTAGGCTGGAGCTGCTTC	CCCCGCCGGAGCGAGGTTTT TTGTTACATCAAAGCGAGAAT TAACGGCTGACATGGGAAT	Integrated DNA Technologies, Inc.
Δ*mltD*::KAN^R	GTTATGATCGGTCGTCTTTT AAGCAACTATTGACACACAC GTGTAGGCTGGAGCTGCTTC	AGGCACCGGGGGAATCGGTG CCTTTTTATTATCTGGTTTGTT AACGGCTGACATGGGAAT	Integrated DNA Technologies, Inc.
Δ*fis*::KAN^R	GAAAATTTTGCGTAAACAGA AATAAAGAGCTGACAGAACT GTGTAGGCTGGAGCTGCTTC	CGAGTAGCGCCTTTTTAATCA AGCATTTAGCTAACCTGAATT AACGGCTGACATGGGAAT	Integrated DNA Technologies, Inc.
Δ*yafO*::KAN^R	ATATGACGGATGATGATTTC AATGACTTTAAGGAATAAGG GTGTAGGCTGGAGCTGCTTC	CGCCAGGCTGATAGTTTCTTAT TTGTATGTTATTCATAATATAA A TTTAACGGCTGACATGGGAAT	Integrated DNA Technologies, Inc.
Δ*guaA*::KAN^R	ATTTATATTATCTGTTTCACT TGCCTCGGAATAAGCGTCA	AGTAACAGAGGGTTTTGTTCAT TCATAGTGCAGGGTCAAATTAA	Integrated DNA

	GTGTAGGCTGGAGCTGCTTC	CGGCTGACATGGGAAT	Technologies, Inc.
Δ*waaO*::KAN^{R}	TACATAAGGTAATTATTTCTGCTATTTCCCGGAGGAAATAGTGTAGGCTGGAGCTGCTTC	CAATGCTACCCTTATATCATTACTTTATAGTTTCCCAGTTTTAACGGCTGACATGGGAAT	Integrated DNA Technologies, Inc.

Oligonucleotides to Verify Gene Deletions

**Mutation**	**External Forward Primer (5' to 3')**	**External Reverse Primer (5' to 3')**	**Internal Forward Primer (5' to 3')**	**Internal Reverse Primer (5' to 3')**	**Source**
Δ*nagA*::KAN^{R}	GTGTGCGATGAACCTTCCAC	GCTCTTCGTCCAGATCATCCT	GGCCACGAATTTCTTGATGAC	CGCAATGTTCGACCAGATTAC	Integrated DNA Technologies, Inc.
Δ*wcaI*::KAN^{R}	CGGATGAGCAGCATGACG	GCTCTTCGTCCAGATCATCCT	CATTACCGCACCGCCTTACTAC	GTCAGTTTCGACGGCAATACG	Integrated DNA Technologies, Inc.
Δ*cpsG*::KAN^{R}	GAGGATGATGTGGTGCGTTTC	GCTCTTCGTCCAGATCATCCT	GCCTATGGCGAATTTCTCAAAC	CGAAATCACGGAAGTAATGGTG	Integrated DNA Technologies, Inc.
Δ*wzxC*::KAN^{R}	CTGCGCCATAAGGTGAAACC	GCTCTTCGTCCAGATCATCCT	CGGATTATCGACAACCACCAG	GTTCCACTTCTCACCAAAGACC	Integrated DNA Technologies, Inc.
Δ*yfcI*::KAN^{R}	GAATTTGCGAGTCAGCTTCC	GCTCTTCGTCCAGATCATCCT	GCGCAAACTGTGTGATTTAACG	CTTTGCGCGATAAGATCGTC	Integrated DNA Technologies, Inc.
Δ*guaB*::KAN^{R}	GTCGATAGTAACCCGCCCTTC	GCTCTTCGTCCAGATCATCCT	CCTCGTTCCTGCTCACTCTACC	CAGCAACAGCGGTAATCTGC	Integrated DNA Technologies, Inc.
Δ*waaG*::KAN^{R}	ATTCTGGGCGGGGAATTATC	GCTCTTCGTCCAGATCATCCT	GTGGGCTTCAACGTGACTTTATG	CAGCGATGACCGTTCCAC	Integrated DNA Technologies, Inc.
Δ*rpmG*::KAN^{R}	CGCACTGAACGCGACTAAAC	GCTCTTCGTCCAGATCATCCT	GGTATTCGTGAGAAAATCAAGCTG	GATTTTCGCTTCTTTGTAGATCACG	Integrated DNA Technologies, Inc.
Δ*mltD*::KAN^{R}	CTGAAGAGCGTTTTGCATGG	GCTCTTCGTCCAGATCATCCT	GCGATATTACTCGCCTCTGTCC	CAGTTGATCTGCATGCTTCTTTG	Integrated DNA Technologies, Inc.
Δ*fis*::KAN^{R}	CAAAAGGGTACCGAATTGCAC	GCTCTTCGTCCAGATCATCCT	CGAACAACGCGTAAATTCTGAC	CAGCGTACCACGGTTGATG	Integrated DNA Technologies, Inc.

Δ*yafO*::KAN^R	GCCCCGCAGG ATATCTCTTA	GCTCTTCGT CCAGATCAT CCT	CAAAACTTATTC GCCTGCAAC	CGAAACGC TTCTGCCA TTTT	Integrated DNA Technologies, Inc.
Δ*guaA*::KAN^R	GCTGTGGTACT ATCGACGAAC TG	GCTCTTCGT CCAGATCAT CCT	CATAAGCATCGC ATCCTCATTC	CAGCGCTT CTTCATCG AATAC	Integrated DNA Technologies, Inc.
Δ*waaO*::KAN^R	GTCAATGGCC ATCTGTATCAA CC	GCTCTTCGT CCAGATCAT CCT	CTGGACATCGCT TATGGAACTG	CTATTGTT CGGTTTCA GCAACG	Integrated DNA Technologies, Inc.

Table S3: Verified mutant strains in *E. coli* MG1655 and BW25113 backgrounds.

Mutant Strains	Strain Background	
Δ*wcaI*	*E. coli* MG1655	*E. coli* BW25113
Δ*cpsG*	*E. coli* MG1655	*E. coli* BW25113
Δ*nagA*	*E. coli* MG1655	*E. coli* BW25113
Δ*wzxC*	*E. coli* MG1655	*E. coli* BW25113
Δ*yfcI*	*E. coli* MG1655	*E. coli* BW25113
Δ*recA*	N/A	*E. coli* BW25113
Δ*guaB*	*E. coli* MG1655	*E. coli* BW25113
Δ*waaG*	*E. coli* MG1655	*E. coli* BW25113
Δ*mltD*	*E. coli* MG1655	*E. coli* BW25113
Δ*rpmG*	*E. coli* MG1655	*E. coli* BW25113
Δ*fis*	N/A	*E. coli* BW25113
Δ*recN*	N/A	*E. coli* BW25113
Δ*yafO*	*E. coli* MG1655	*E. coli* BW25113
Δ*guaA*	*E. coli* MG1655	*E. coli* BW25113
Δ*waaO*	*E. coli* MG1655	*E. coli* BW25113

Table S4: Concentrations of bactericidal antibiotics used in persister assays.

Bacterial Strain	Bactericidal Antibiotics	MIC Range (µg/ml)	Persister Assay Concentration (µg/ml)
Escherichia coli K-12 MG1655 WT	Ampicillin	3.125-6.25 (determined in our previous study) (4)	200
Escherichia coli K-12 MG1655 WT	Ofloxacin	0.039-0.078 (determined in our previous study) (4)	5
Escherichia coli K-12 MG1655 WT	Ciprofloxacin	0.03 (this study)	3
Escherichia coli K-12 MG1655 WT	Gentamicin	0.5	50
Escherichia coli K-12 MG1655 WT	Fosfomycin	0.5 (5)	300

References

1. Keseler IM, Mackie A, Santos-Zavaleta A, Billington R, Bonavides-Martínez C, Caspi R, Fulcher C, Gama-Castro S, Kothari A, Krummenacker M, Latendresse M, Muñiz-Rascado L, Ong Q, Paley S, Peralta-Gil M, Subhraveti P, Velázquez-Ramírez DA, Weaver D, Collado-Vides J, Paulsen I, Karp PD. 2017. The EcoCyc

database: reflecting new knowledge about Escherichia coli K-12. Nucleic Acids Res 45:D543–D550.

2. Orman MA, Brynildsen MP. 2013. Dormancy is not necessary or sufficient for bacterial persistence. Antimicrob Agents Chemother 57:3230–3239.

3. Zaslaver A, Bren A, Ronen M, Itzkovitz S, Kikoin I, Shavit S, Liebermeister W, Surette MG, Alon U. 2006. A comprehensive library of fluorescent transcriptional reporters for Escherichia coli. Nat Methods 2006 38 3:623–628.

4. Mohiuddin SG, Hoang T, Saba A, Karki P, Orman MA. 2020. Identifying Metabolic Inhibitors to Reduce Bacterial Persistence. Front Microbiol 0:472.

5. Amato SM, Brynildsen MP. 2015. Persister Heterogeneity Arising from a Single Metabolic Stress. Curr Biol 25:2090–2098.
